# Chirality Detection in Scanning Tunneling Microscopy Data Using Artificial Intelligence

**DOI:** 10.1002/smtd.202400549

**Published:** 2024-09-09

**Authors:** Tim J. Seifert, Mandy Stritzke, Peer Kasten, Björn Möller, Tim Fingscheidt, Markus Etzkorn, Timo de Wolff, Uta Schlickum

**Affiliations:** ^1^ Institute of Applied Physics TU Braunschweig 38106 Braunschweig Germany; ^2^ Institute of Analysis and Algebra TU Braunschweig 38106 Braunschweig Germany; ^3^ Institute for Communications Technology TU Braunschweig 38106 Braunschweig Germany; ^4^ Laboratory for Emerging Nanometrology TU Braunschweig 38106 Braunschweig Germany

**Keywords:** chiral networks, molecular self‐assembly, machine learning, object detection, scanning probe microscopy, synthetic training data

## Abstract

Enantiospecific effects play an uprising role in chemistry and technical applications. Chiral molecular networks formed by self‐assembly processes at surfaces can be imaged by scanning probe microscopy (SPM). Low contrast and high noise in the topography map often interfere with the automatic image analysis using classical methods. The long SPM image acquisition times restrain Artificial Intelligence‐based methods requiring large training sets, leaving only tedious manual work, inducing human‐dependent errors and biased labeling. By generating realistic looking synthetic images, the acquisition of real datasets is avoided. Two state‐of‐the‐art object detection architectures are trained to localize and classify chiral unit‐cells in a regular molecular chiral network formed by self‐assembly of linear molecular bricks. The comparison of different architectures and datasets demonstrates that the training on purely synthetic data outperforms models trained using augmented datasets. A Faster R‐CNN model trained solely on synthetic data achieved an excellent mean average precision of 99% on real data. Hence this approach and the transfer to real data show high success, also highlighting the high robustness against experimental noise and different zoom levels across the full experimentally reasonable parameter range. The generalizability of this idea is demonstrated by achieving equally high performance on a different structure, too.

## Graphical abstract

The lack of available annotated Scanning Probe Microscopy (SPM) data limits the applicability of Machine Learning to evaluate images on the nanoscale, requiring laborious manual work. The use of synthetic data to train state‐of‐the‐art object detection models can overcome this limitation, enabling a fast, accurate, and reliable analysis of SPM images, as demonstrated on two self‐assembled molecular chiral networks.

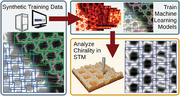

## 1 Introduction

The local alignment and conformation of organic molecules at surfaces is key for designing and controlling highly functional molecular systems. Since tiny changes in the local positioning may influence the molecules chemical and physical properties tremendously, chirality adds another variance of tuning their characteristics. Nowadays, chiral organic networks attract significant interest in the field of heterogeneous catalysis,^[^ ^1^, ^2^, ^3^, ^4^, ^5^, ^6^ ^]^ non‐linear optics,^[^ ^7^, ^8^, ^9^ ^]^ and enantiospecific sensing.^[^ ^10^, ^11^, ^12^, ^13^ ^]^ Since the functionality of chiral organic networks depends on their handedness, a fast evaluation method to determine the chirality of each unit cell is inevitable to enhance optimization mechanisms. Scanning tunneling microscopy (STM) is one of the most important tools to probe the atomistic arrangement of molecular surface structures. While the imaged molecules and networks of molecules can be identified manually, this can lead to human‐depending biases in labeling and can be very time consuming.

These problems can be overcome by machine learning (ML), which can identify structures automatically using object detection to categorize, localize, and count objects. This has already been widely encountered by using neural networks and was part of the work of multiple groups in surface sciences as well.^[^ ^14^, ^15^, ^16^ ^]^

However, to detect the desired features, a ML algorithm needs to be trained. A key problem in training a neural network is to collect sufficient amounts of labeled training data. Thus, a big dataset of real images is needed and these images have to be labeled. Typically, in scanning probe microscopy (SPM), such big datasets are not available due to the high acquisition time of each image. Moreover, it is enormously time consuming to label a large number of real images manually.^[^ ^15^, ^17^ ^]^ Hence, the lack of available labeled real SPM image data is often a limiting factor in the implementation of artificial intelligence (AI) in image analysis procedures.

For different applications of SPM and Scanning Transmission Electron Microscopy, AI has been incorporated widely, reaching from autonomous scanning^[^ ^15^, ^18^, ^19^ ^]^ over atomic site identification^[^ ^20^ ^]^ and image enhancement^[^ ^21^, ^22^ ^]^ to specific AI frameworks^[^ ^23^, ^24^ ^]^ and databases.^[^ ^25^, ^26^, ^27^ ^]^ In multiple approaches, AI‐based object detection has been used to find interesting regions or image defects with the aim to automate the image acquisition^[^ ^15^, ^19^ ^]^ or tip shaping processes.^[^ ^17^, ^19^ ^]^

Nevertheless it has rarely been applied for structural analysis of SPM data. Only one example in the detection of chiral molecules has been proposed using just one manually labeled real image of good quality to generate a large training set based on a variety of augmentation techniques to avoid the need for a large labeled real dataset.^[^ ^14^ ^]^ But as discussed in detail later, this method is limited to special cases only.

An approach to overcome the need for any manual labeling is to create synthetic images.^[^ ^28^, ^29^ ^]^ This has the advantages of fast creation speed and automatically perfect labels. But synthetic images must be as close as possible to real images to ensure that a network trained on synthetic data performs well in evaluating real data, too.

**Figure 1:**
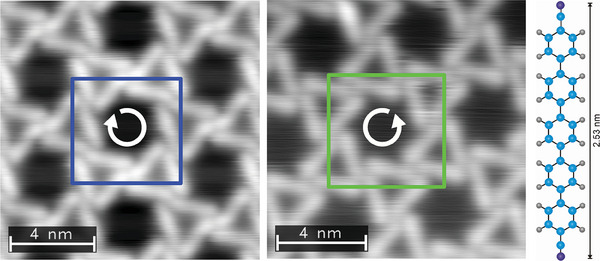
Two images of NC − Ph_5_ − CN on Ag(111) assembling in clockwise (blue, left image) and counterclockwise (green, middle image) chirality. The image to the right shows the atomic composition of NC − Ph_5_ − CN.^[^ ^30^ ^]^

In this article, we automatize and simplify the analysis of chiral networks using ML trained on synthetic data, aiming to drastically reduce the examination time. We illustrate our approach on the exemplary structure shown in **Figure** **1**, a chiral kagomé lattice formed by the self‐assembly of dicarbonitrile‐pentaphenyl (NC − Ph_5_ − CN) upon adsorption on Ag(111).^[^ ^30^, ^31^, ^32^, ^33^, ^34^, ^35^, ^36^ ^]^ The structure is hierarchical: Three molecules form a triangle and six triangles arrange in an open hexagon with a size of about 6 nm. This network is formed by achiral linear molecular bricks and the chirality is solely introduced by the clockwise or anticlockwise arrangement of the triangular units building the kagomé lattice. The real images have been acquired for a wide range of zoom levels (image edge lengths of 10 – 292 nm), which we aim to analyze with a single network. We prefer this One‐fits‐most approach over separate networks to simplify the application.

To solve the issue caused by the low number of real images, we present a framework to generate realistic‐looking synthetic data, train our network on this dataset and evaluate its ability to perform the detection on real STM images. In total, we compare eight different models that differ by the data generation technique and the model structure.

We substantially extend existing approaches based on augmented data in both, the generation of training data, as well as the AI framework. This strongly increases the robustness of the method and leads to a high performance for a much broader dataset range regarding the quality and zoom level of the images, as well as its transferability to different structures.

## 2 Experimental Section

### 2.1 Synthetic Training Data

**Figure 2:**
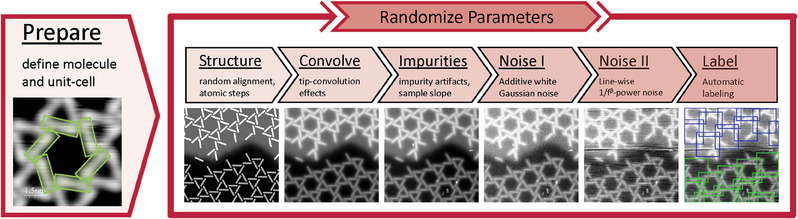
Generation steps for synthetic images. After the appearance of the molecule and its unit‐cell is defined once, synthetic images are generated by a random alignment of the kagomé lattice with respect to the image axes, further modified by step edges. Realism is improved by including tip‐convolution effects, impurities and a slope, white noise, and 1/*f* ^*b*^‐noise. Additionally, labels for object detection tasks are visualized.

To eliminate the most crucial limitation of applying AI to STM tasks, a set of purely synthetic data was generated whose depicted area covers a wide size‐range, occurring as apparent zoom levels in the images. The generation consisted of multiple steps visualized in **Figure** **2**: In preparation to generate a new set of synthetic data, the basic shape of the molecule had to be defined, which was here simply modeled as a rectangle of certain width and height. Additionally, the relative positions and angles of molecules need to be defined to specify the unit‐cell. This prior knowledge can simply be determined by available experimental images or theoretical calculations. The pre‐defined lattice structure was randomly aligned on the surface with randomized alternations in molecular positions and angles. Additionally, the structures were varied nearby atomic steps. This simple geometric model served as the base to generate realistic images in multiple steps: Tip‐convolution effects were include by switching the rectangular height profile of molecules to a sigmoid function. Like real images, these images were tilted along a slope, and impurities were added. Experimentally realistic noise was modeled as additive white Gaussian noise, as well as line‐wise noise. Since the molecular structure was well‐known, labels could be generated for many ML tasks, most importantly bounding boxes for object detection, as visualized in the last step of Figure 2. The bounding boxes were always aligned along the image axes, with their width and height defined to contain the entire hexagon. Oriented object detection, rotating the box according to the structure, was not applicable due to the rotational symmetry of the object. With these modifications, a great variety of images could be generated.

The hexagon‐like ring of six molecules was chosen to be the object to identify in all datasets, since its larger size reduces computational effort for images with low zoom showing many objects compared to detecting the small triangles. The targeted bounding boxes are shown in Figure 2. The software can be customized easily to replicate different molecular networks or labels; further information is provided in the Section S3 (Supporting Information).

Since the area scanned by STM for each image was chosen freely in the experiment, it was necessary to distinguish between the zoom level, which was defined as the edge length of the real quadratic imaged area measured in nanometer, and the resolution of the image, given by the number of pixels in each image, in this case fixed to 512 × 512 px. For the ability to resolve and evaluate the substructure of the unit‐cell, only the number of pixels per unit‐cell was relevant, meaning that images of very large scale could be detected as accurately as small‐scale images, if just the number of pixels used to capture the image would be increased accordingly. Therefore, it should be kept in mind that the number of pixels can always be increased to ensure good performance on large‐scale images, albeit with higher experimental effort.

### 2.2 Object Detection Setups

In this study, the goal was to investigate the influence of different methods for generating training data, as well as different model architectures on analyzing synthetic and real images. The analysis setup was therefore structured into multiple stages. Each stage consisted of one out of four specific model architectures and was trained on one of two sets of training data. As a result, eight different scenarios were obtained, each with a unique combination of architectural setup and the data it was trained on. By successively changing the model parameters in each stage, the effect of each modification could be determined. The initial focus was on the evaluation of pure synthetic generated images to guarantee fair comparison. Two different types of training datasets were generated.

(1) One single labeled image was chosen and heavy image augmentation was applied to generate a large number of images for training the networks.^[^ ^14^ ^]^ This will be referred to as dataset “Aug”.
(2) The presented method was used to generate many different synthetic images that include all kinds of zoom levels and qualities to train the networks on. The set will be referred to as dataset “Synth”.
  The two datasets were used to train four different model architectures. As a baseline method, the Faster Region‐based Convolutional Network (Faster R‐CNN) model setup provided by Li et al.^[^ ^14^ ^]^ was used. From there on, changes were successively applied to the model architecture to probe performance gains (see **Table** **1**).
  (I) The exact settings as provided by Li et al. were used, which also include cropping procedures inside the network: Training images were cut into pieces to be trained on individually, therefore improving the recognition of different sized objects. Early stopping was used after 16 epochs to avoid overfitting. This Faster R‐CNN setup will be referred to as model *F*, with an additional index for the respective training datasets “Synth” and “Aug”.
  (II) Two additional smaller anchors were incorporated, so the network searches for smaller objects and gains the capacity to effectively detect them (see Section S4, Supporting Information). Since the dataset “Synth” was already very diverse regarding zoom levels and covers a wide range of pixels per unit cell, cropping was no longer applied inside the network. This results in stage F*.
  (III) The same model setup as before was used, but instead of training on 1000 images, the size of the training set was increased to 10 000 images. The number of training epochs was adjusted from 16 to 1.6, as well to ensure that the number of training steps stays the same, they only arise from a larger dataset. This stage will be referred to as model $F_{10k}^{*}$.
    These models, which were all based on the same Faster R‐CNN architecture were compared with another state‐of‐the‐art network architecture YOLO (“You Only Look Once”) as the fourth experimental stage.
  (IV) The YOLOv5 architecture of medium size^[^ ^37^ ^]^ was trained for an equivalent time duration as the Faster R‐CNN networks, translating to 30 epochs using 10 000 images for both datasets and denote this stage as *Y*. YOLOv5 was preferred over YOLOv8 due to its faster training speed, but the results using YOLOv8 will be discussed later.

In total, three Faster R‐CNN setups and one YOLO setup were compared, with each trained on both datasets, “Aug” and “Synth”. This constitutes a total of eight scenarios to be compared (see Table 1). Details on model setups and parameters are given in Section S4 (Supporting Information). All models were trained on an NVidia GTX 1080 Ti GPU.

**Table 1:** Overview of the different models: The experiments are separated in four stages, which differ in the model architecture, the number of epochs the model has been trained for, the number of training images, and changes in the model parameters.

Stage	Architecture	Dataset size	Epochs	Modifications
*F*	Faster R‐CNN	1000	16	Baseline settings^[^ ^38^ ^]^
F*	Faster R‐CNN	1000	16	Smaller anchors, no cropping (see Section S4, Supporting Information)
$F_{10k}^{*}$	Faster R‐CNN	10 000	1.6	Increase training set size
*Y*	YOLOv5	10 000	30	Usage of YOLO architecture (see Section S4, Supporting Information)

### 2.3 Metrics

The performance of the models was evaluated quantitatively, based on a couple of metrics: The precision *P* determines the percentage of correct predictions out of all predictions, the recall *R* determines the percentage of correct predictions out of all objects in the images (see Section S5, Supporting Information for details). For each prediction, the model also estimates a confidence to quantify the certainty of each particular detection. Varying the confidence threshold and plotting the precision over the recall yields the precision‐recall curve, examples are shown in Section S6 (Supporting Information). The area under the precision‐recall curve was the most important metric, the mean average precision mAP (0 ⩽ mAP ⩽ 1) with higher values indicating better performance.^[^ ^39^ ^]^ This metric was defined for a given image size, thus number of pixels per unit cell. To evaluate the quality of the methods for different zoom levels, the average mAP was introduced, denoted as 〈*mAP*〉, which assured that each zoom level contributes equally to the performance score. Details on the calculation of 〈mAP〉 are given in the Section S5 (Supporting Information).

## 3 Results and Discussion

### 3.1 Evaluation of synthetic data

#### 3.1.1 Quantitative Analysis

In **Table** **2**, we present the 〈mAP〉 results for all models and datasets, evaluated on synthetic data. The baseline model *F*_*Aug*_ demonstrates a low performance level, achieving 0.237 in 〈mAP〉. When training the same model on the dataset “Synth” generated by our method, we observe a moderate improvement, with the metric increasing to 0.562. With our adjustments to the Faster R‐CNN (F*) and “Synth” training data, the performance improves remarkably, reaching a 〈mAP〉 of over 0.9. A larger training set size ($F_{10k}^{*}$) increases the performance only marginally. However, adopting the YOLO architecture leads to an additional slight improvement reaching a 〈mAP〉 of almost 0.94. The time to compute the predicted bounding boxes for each image, the inference time, is reasonably fast and stays beneath 300 ms for each stage as listed in Section S7 (Supporting Information).

**Table 2:** 〈mAP〉 for all four setups and both training datasets, trained on either “Aug” or “Synth”, evaluated on the dataset “Synth”.

Stage	“Aug” training data	“Synth” training data
*F*	0.237	0.562
F*	0.178	0.911
$F_{10k}^{*}$	0.173	0.931
*Y*	0.320	0.938

#### 3.1.2 Visual Analysis

**Figure 3:**
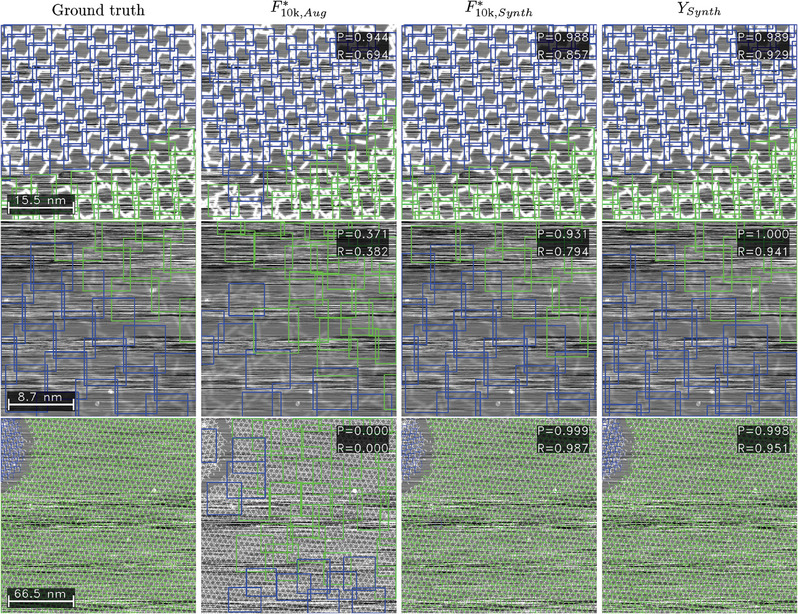
Example results for synthetic images. The first column shows the ground truth (GT), the second column the model $F_{10k}^{*}$ trained on dataset “Aug”, and third and fourth columns the results from stages $F_{10k}^{*}$ and *Y* using dataset “Synth”. The first row shows an example image of size 47 nm, the second row a noisy image of size 26 nm, and the last row a large scale image with a size of 199 nm. The confidence threshold for all predictions is 0.7.

In **Figure** **3**, we visualize the results discussed on specific examples. The columns of the figure show the ground truth (labels) and the predictions by the models $F_{10k,Aug}^{*}$, $F_{10k,Synth}^{*}$, and *Y*_*Synth*_ at a confidence threshold of 0.7 to cover a sufficiently large range of plausible predictions for the visual evaluation. In the first row, we consider an image with a size of 47 nm, which is close to the size of the initial training image used to generate dataset “Aug”. Here, all three networks demonstrate reasonable detection capabilities. YOLO shows highest precision and recall; $F_{10k,\mathrm{Aug}}^{*}$ shows a higher number of false‐positive detections compared to YOLO but still gets a competitive precision.

In the second row, we investigate an image of size 26 nm with a considerably high noise level. The $F_{10k}^{*}$ model trained on dataset “Aug” (second column) yields some correct predictions, but leads to various false positives while missing to detect numerous other objects entirely. Contrarily, both models trained on dataset “Synth” demonstrate very strong detection capabilities.

Finally, in the last row, an image with a size of 199 nm is presented in which the unit cell only consists of very few pixels. The behavior differs drastically between the datasets: Faster R‐CNN trained on dataset “Aug” (second column) performs significantly worse than both models trained on “Synth” data: It yields various predictions, which are not only all false, but even have box sizes, which do not match the sizes of the depicted objects. The initial image for the augmentation method was required to have both chiralities present in one image. For our use case, the unit cells of both chiralities are spatially separated. As a result, the models tend to project the learned spatial distribution from the training image onto new inputs. Thus, the model is biased due to the lack of variance in the training data, which is called overfitting and a frequently occurring obstacle in the training of ML models. For both models trained on dataset “Synth”, this is not the case, resulting in excellent precision and recall scores above 0.95.

#### 3.1.3 Dependence on Zoom Level

**Figure 4:**
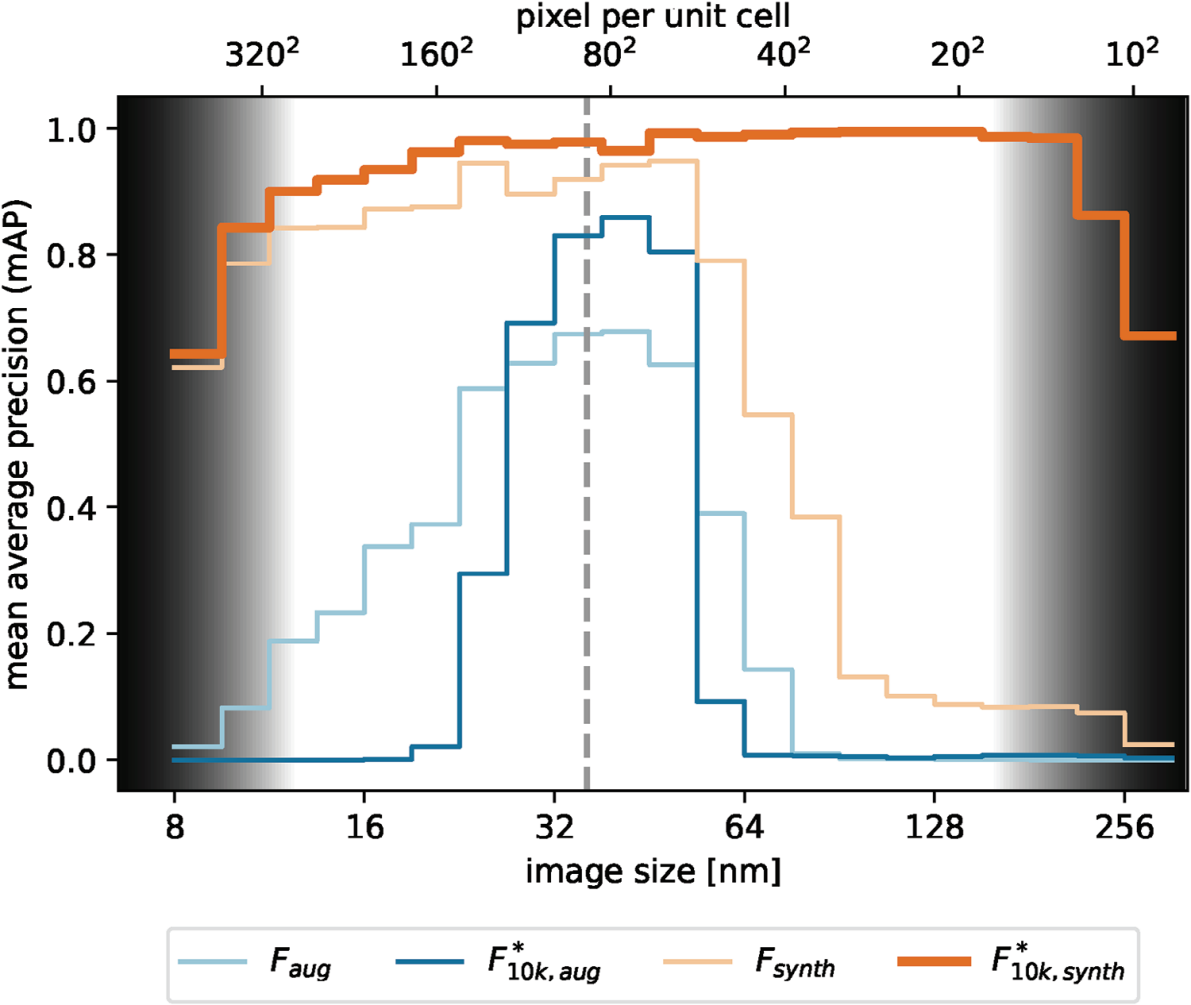
Mean average precision per image size for selected models evaluated on the dataset “Synth”. The size of the input image to generate dataset “Aug” is shown as a dashed line. Gray regions indicate the area in which constraints of the experimental setup become important, limiting the achievable performance independently of the models themselves.

This result indicates that especially for large images the performance differs strongly according to the used training dataset. For further analysis, we evaluate synthetic images across all probed zoom levels, and quantify the results of images of different zoom separately. **Figure** **4** provides a graphical representation of the performance metric of selected models for zoom levels from 8 to 300 nm. To preserve clarity, we only show the stages *F* and $F_{10k}^{*}$ trained on both datasets.

The size dependency of the mAP substantially differs between the models trained on dataset “Aug” compared to those trained on dataset “Synth”: For both models trained on augmented data, the range of good performance is concentrated only around the size of the image used as a training input, which is in this case 36 nm, indicated in Figure 4 as a dashed line. Around 36 nm, the models show reasonably good performance and achieve their peak mAP of 0.68 for model *F*_*Aug*_ and 0.86 for model $F_{10k,Aug}^{*}$, which drops off rapidly to both sides. The graph for model $F_{10k,Aug}^{*}$ reaches a higher peak performance, but over a much smaller range of pixels per unit cell compared to model *F*_*Aug*_. This is a consequence of the modifications from model *F* to model $F_{10k}^{*}$: Removing the cropping procedures reduces the apparent size flexibility of the model. This results in a setup, which is specialized to the size of the initial training image and reaches higher accuracy in this range, but cannot recognize images that differ in size due to the low variability in the training data. Throughout all setups using augmented training data, the more the zoom level differs from the initial training image, the worse the models perform, reaching good performance only in a narrow region. These models therefore do not support the One‐Fits‐Most idea we pursue in this work, aiming to handle images of various image sizes with the same network. Additional tests have proven that the model *F* is limited by its structure and not by the reduced number of training images (see Section S8, Supporting Information).

Contrarily, for the same models trained on dataset “Synth”, the graphs show a different behavior. The performance is much higher than for “Aug” models and stays high for a much wider range of zoom levels. While model *F*_*Synth*_ exhibits a peak mAP above 0.9 for images from 25 to 50 nm, model $F_{10k,Synth}^{*}$ demonstrates exceptional performance over a significantly wider range from 20 to 215 nm with an mAP above 0.95. This highlights the improvements obtained by our modifications to the baseline architecture, including smaller anchors, as well as removing cropping procedures. By introducing an intermediate step, we separate the performance gains from model *F* to F* into both contributions. From this, it can be concluded that most of the improvement stems from the changed anchor sizes, see Section S8 (Supporting Information).

Toward small images, both “Synth” models behave similarly: The performance drops for images smaller than 10 nm. This can be explained by the sensitivity of mAP at low numbers of objects: At these sizes, only very few (⩽5) unit cells are present and edge effects caused by only partially displayed objects in the image arise, yielding apparently worse results in mAP (for an example see Section S9, Supporting Information).

The drop of performance for images above 215 nm for model $F_{10k,Synth}^{*}$ arises purely from experimental constraints: At a resolution of 512 × 512 px, the number of pixels per unit cell is not sufficient to resolve the chiral structure anymore, making separation of both chiralities even by eye impossible (see Section S10, Supporting Information).

The models *Y* and F* trained on dataset “Synth” exhibit only minimal differences to $F_{10k}^{*}$, hence they are not depicted in Figure 4. Instead, we compare models $F_{10k}^{*}$ and *Y* in Section S6 (Supporting Information), indicating a different behavior for high confidence thresholds, only.

#### 3.1.4 Disucssion: Synthetic Data

To conclude the results on synthetic data, we observe that the only hard limitation for our approach is determined by real world effects and lies beyond the influence of our chosen model: the number of pixels per unit cell, which is set by the image resolution in the experiment. In consequence, the restriction that the performance is limited to images of size smaller than 215 nm for synthetic images holds true only for our chosen experimental image resolution of 512 × 512 px. If we chose, for example, images with a pixel size increased by a factor of 1.5 to 768 × 768 px, we observe a drop in performance also by a factor of 1.5 later, thus around 325 nm, see Section S11(Supporting Information). It follows that the observed drops in performance for optimized models trained on synthetic data are mainly a direct consequence of the experimental setup and are not due to the limits of the models themselves. The areas of experimental limitations are indicated by the gray regions in Figure 4.

Both models, Faster R‐CNN and YOLO, trained on our generated synthetic dataset perform very well and support our One‐Fits‐Most‐Idea. While the performance of both architectures is similar, YOLO has advantages regarding its usability, since it performs better using default model parameters and consumes less computational power, especially regarding the memory requirements.

### 3.2 Evaluation of Real STM Images

#### 3.2.1 Quantitative Analysis

In addition to the evaluation of synthetic images, we evaluate real images in which we hand‐labeled the data. In contrast to synthetic images, we evaluate real images only based on the regular mAP value. An obtained 〈mAP〉 value would not be meaningful, since the physical sizes of real images are not evenly distributed in the dataset.

For some setups, we observe a significant variation in performance depending on the zoom level and quality of the image. Therefore we partition our data into “normal” and “hard” images, which are heavily influenced by experimental noise or defects to a point that even manual investigation is not trivial anymore (for examples see Section S12, Supporting Information). We also split off a set of large images and evaluate the performance on this set separately. Before the models are applied to real data, standard automatic preprocessing is performed, such as line‐corrections and contrast enhancement. This step however improves the performance just slightly (see Section S13, Supporting Information). We present a summary of mAP values for each experimental stage in **Table** **3**.

**Table 3:** Evaluation of the real images with the different models.

Stage	Dataset “Aug”			Dataset “Synth”		
	mAP_<80 nm_	mAP_⩾80 nm_	mAP_hard_	mAP_<80 nm_	mAP_⩾80 nm_	mAP_hard_
*F*	0.347	0.000	0.064	0.904	0.074	0.132
F*	0.552	0.004	0.044	0.988	0.934	0.696
$F_{10k}^{*}$	0.531	0.003	0.036	0.991	0.865	0.673
*Y*	0.344	0.005	0.035	0.944	0.793	0.561

Considering real images with a size beneath 80 nm, we observe a large gap between the models trained on datasets “Aug” and “Synth”: For augmented training data, we reach a mAP of around 0.35 for the stages *F* and *Y*. The adaptions to the Faster R‐CNN architecture and training set size were able to increase the performance up to 0.55 (F*) and 0.53 ($F_{10k}^{*}$). By switching to training dataset “Synth”, the performance was improved drastically, reaching values between 0.9 for stage *F* and up to 0.99 for stage $F_{10k}^{*}$. The mAP obtained by the YOLO architecture in combination with synthetic training data stays with 0.94 behind the modified Faster R‐CNN, albeit still with an exceptional performance (Table 3).

#### 3.2.2 Visual Analysis

**Figure 5:**
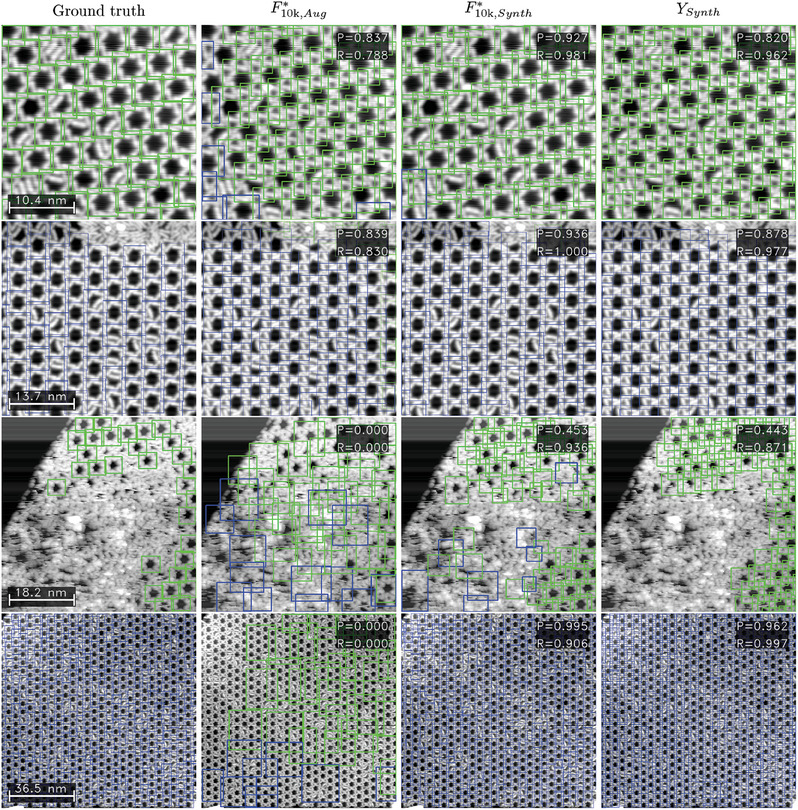
Example results for real images. The first column shows the ground truth (GT), the second column model $F_{10k}^{*}$ trained on dataset “Aug”, and the third and fourth columns the results from stages $F_{10k}^{*}$ and *Y* using dataset “Synth”. The examples show images of size 31 and 41 nm in the first two rows, a “hard” image with size 55 nm, and a “large” image of size 110 nm. The confidence threshold for all predictions is 0.7.

Some examples on individual real images are shown in **Figure** **5** to demonstrate how the detection process of the chiral unit cell performs using the different models. The first two rows show two images of the kagomé structure with a size of 31 nm in counterclockwise and a size of 41 nm in clockwise chirality. Since the size of these images is close to the size of the initial training image for dataset “Aug” (36 nm), model $F_{10k}^{*}$ trained on this dataset achieves considerably high precision and recall values around 0.8. It stands out that false predictions are especially located at the edges, following the spatial distribution of chiralities present in the training image. The models trained on dataset “Synth” show even higher localization performance, reaching a high precision and near‐perfect recall.

When evaluating images categorized as “hard”, all “Aug” models fail, reaching mAP values below 0.1 (Table 3). This is also underlined by the exemplary image in the third row of Figure 5. In contrast, even on real images with excessive noise that are hard to identify for the human eye, the models trained on synthetic data give good predictions. Model F* achieves an mAP of almost 0.7, which falls behind the results for images of good quality, but can still be interpreted as a remarkable result given the image quality. As seen in the third row of Figure 5, the recall of both models $F_{10k}^{*}$ and *Y* is still very high at 0.94 and 0.87 respectively, albeit with drawbacks in the precision.

#### 3.2.3 Dependence on Zoom Level

The findings gained by analyzing the performance of synthetic images with different zoom levels carry on to the real data, too. Throughout all example images, it stands out that all models trained using dataset “Aug” are biased due to the lack of variance in the training data. Therefore, they fail in evaluating real images larger than 80 nm with mAP values below 0.01. This overfitting behavior, already observed for the evaluation of synthetic images, becomes especially apparent for the hard and large images: The model $F_{10k,Aug}^{*}$ artificially forces the presence of both classes, resulting in predictions that seem arbitrary, both in box size, localization and class assignment (see Figure 5).

#### 3.2.4 Discussion: Real Data

If a model, Faster R‐CNN or YOLO, is trained on “Synth” training data, no overfitting behavior and limitations on the image size over a wide range occur, as they do not depend on a single image. On images larger than 80 nm model F* emerges as the best stage with a mAP of over 0.93. Models $F_{10k}^{*}$ and *Y* perform slightly worse, but still deliver excellent performance as becomes apparent in Figure 5. We see similar constraints as for the evaluation on synthetic data arise at very small and very large images due to the low number of objects, or the low number of pixels per unit cell, respectively. Imaging at higher resolution could expand the performance to larger images, although we point out that further noise effects might occur depending on the experimental setup.

In contrast to the findings on synthetic data for which the YOLO model achieved best performance, the Faster R‐CNN architecture delivered best results on real data. This indicates that in our use case YOLO is more specialized to the synthetic images, while Faster R‐CNN provides better generalization.

In summary, the training based on the generation of purely synthetic data achieves a high success rate for the detection of unit cells in both, synthetic and real images. Our method to create synthetic data offers high flexibility in providing training datasets, which are very broad in terms of image quality and image size, enabling robust training of our models with datasets of sizes and variability hard to be captured by real STM imaging and manual labeling due to time constraints. Specifically, synthetic data generation allows to generate images over a wide range of pixels per unit cell, enabling the training of AI for an extensive variety of apparent zoom levels.

#### 3.2.5 Effects of Longer Training

As a final step, we aim to probe potential further performance gains if we train the models without early stopping. Apart from the most advanced Faster R‐CNN stage $F_{10k}^{*}$ and the YOLOv5 model, we also include the latest version, YOLOv8. This model comes with higher computational effort, thus the results after the designated time have been substantially weaker than for YOLOv5. If we however train YOLOv8 for longer, we indeed see a significant improvement over YOLOv5 (see **Table** **4**).

**Table 4:** Evaluations of models trained without early stopping.

Stage	Synthetic data	Real data		
	〈mAP〉	mAP_<80nm_	mAP_⩾80nm_	mAP_hard_
$F_{10k}^{*}$	0.943	0.991	0.877	0.689
YOLOv5	0.956	0.969	0.815	0.596
YOLOv8	0.980	0.988	0.923	0.641

The performance of the Faster R‐CNN stage improved just barely with an increased training duration by a factor of 2.5, leading to the same mAP for small images, and a negligible increase for large and hard images of around 2%. Further training leads to worse results on real data. Training YOLOv5 for up to 100 epochs lead to an improvement for all reported metrics, reaching the now highest 〈mAP〉 on synthetic data, but still falling behind other models on real data (see Section S14, Supporting Information). Finally, a YOLOv8 network was trained for 30 epochs, the same as the regular YOLOv5 stage, but taking about 50% longer to train. This model now performs even better than the longer trained YOLOv5 model, reaching a near perfect 〈mAP〉 of 0.98, improving the results especially for very large and very small images. The performance on real data is also significantly increased over YOLOv5, reaching almost the capabilities of stage F* with an equal mAP on small, and slightly worse mAP on large and hard real images (see Table 3). For model F*, longer training did not improve the results. Training YOLOv8 for up to 100 epochs improves the performance on synthetic data, but for real data, the results get worse. Further insights on the performance of YOLOv8 are discussed in Section S15 (Supporting Information).

To conclude, model F* still performs best on real data. Compared to the baseline Faster R‐CNN however, YOLO models work significantly better with default parameters and deliver convincing results. When accounting for time efficiency, YOLOv5 outperforms YOLOv8 on real data by a significant margin, but if the training time is extended, YOLOv8 stands out as the best solution, almost reaching the capabilities of model F* on real data.

### 3.3 Transfer to Different Chiral Network

**Figure 6:**
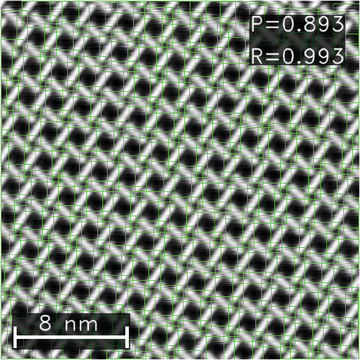
Results on a real exemplary image of NC − Ph_4_ − CN. The image has a size of 26 nm. The confidence threshold is 0.7.

To illustrate that our approach, consisting of synthetic data generation and training of an object detection network, is able to tackle more than one specific system, we have investigated another chiral organic network. The basis for this second molecular lattice is dicarbonitrile‐tetraphenyl (NC − Ph_4_ − CN), the same molecule as before, except one missing phenyl group. Despite a similar basic building block, the resulting network is very different and consists of simpler, open, chiral rhombic units (see **Figure** **6**) with a unit cell almost half as large as for NC − Ph_5_ − CN.^[^ ^30^, ^31^, ^32^ ^]^ Since the unit cell is much smaller, many more objects need to be detected for the same image size compared to the kagomé structure. This imposes high memory requirements for Faster R‐CNN, especially for larger images. As observed during experiments, YOLO has benefits over Faster R‐CNN regarding memory requirements and handles both kagomé datasets better without any modifications to the model structure and parameters, providing better usability. Hence, only the YOLO model is used to evaluate this other chiral structure.

On synthetic data, the model achieved a 〈mAP〉 of over 0.93 for images of up to 108 nm. We find a similar performance drop at the same pixel/unit cell value than for NC − Ph_5_ − CN. Since the unit cell is much smaller, this however translates to a smaller image size. Similarly to Figure 4, we show the size‐dependence of the mAP in the Section S17 (Supporting Information). On real data, the performance drops correspondingly to what has been observed before. For real images with image sizes of less than 108 nm, the mAP reaches 0.89, on images labeled as “hard” it still achieves a mAP of 0.85. An exemplary result on real data is shown in Figure 6, which also indicates the greater challenges for this task compared to the kagomé lattice: Since the unit cell is smaller and each molecule is assigned to two objects, the bounding boxes strongly overlap, which is more demanding for the detection network, since the correspondence between an object and its box is not as clear anymore. Nevertheless, the detection results are still near‐perfect with a precision of 0.89 and a recall of 0.99.

We proved that the applicability of our approach is very flexible, both using Faster R‐CNN and YOLO. It is mostly a user's preference, which of both to apply.

## 4 Conclusion and Outlook

In this article, we compare an existing approach for detecting STM‐imaged molecular chiral networks using augmented training data^[^ ^14^ ^]^ with a novel approach based on the generation of synthetic data and the usage of Faster R‐CNN and YOLO networks. The approach proposed here leads to substantial improvements, showing excellent performance on both, synthetic and real data. It analyzes chiral networks using purely synthetic training data, leading to a peak mAP performance on good real scanning probe microscopy data of ≈ 99% on small and over 93% on large image sizes. This result outperforms existing methods and allows us to analyze large amounts of data in negligible time without introducing any bias by manual evaluations. Moreover, it analyzes molecular networks measured on any zoom level, serving as a *One‐fits‐most* model. Additionally, we demonstrated a successful transfer to a different chiral structure emphasizing the general applicability of our setup to new structures. In perspective, our approach can be used to extract statistically relevant information from the data (for example see Section S16, Supporting Information), which would not be accessible by manual analysis due to time constraints. This enables a quick, reliable and automated evaluation of various enantiospecific catalytic processes, which is essential for future technical advances. In our opinion, this approach can be generalized much further. A manifest next research step is the generalization to various other molecular structures.

## Conflict of Interest

The authors declare no conflict of interest.

## Author Contributions

T.S. and M.S. contributed equally to this work. T.d.W., T.F., M.E., and U.S. conceived this project. T.d.W. and U.S. coordinated this project. T.S. developed the method to generate training data. M.S. defined the machine learning setup with support from B.M and the models have been trained by M.S and P.K.. M.S., P.K., and T.S. evaluated the results. All authors participated in discussions. The first draft of the manuscript was written by T.S., M.S.. All authors contributed to the editing of the manuscript.

## Supporting information

Supporting Information

## Data Availability

The data that support the findings of this study are openly available in Chirality Detection in STM via Artificial Intelligence at https://moto.math.nat.tu‐bs.de/appliedalgebrapublic/chiralitydetectioninstmviaartificialintelligence.

## References

[1] M. O. Lorenzo , S. Haq , T. Bertrams , P. Murray , R. Raval , C. J. Baddeley , J. Phys. Chem. B 1999, 103, 10661.

[2] M. Heitbaum , F. Glorius , I. Escher , Angew. Chem., Int. Ed. 2006, 45, 4732. 10.1002/anie.200504212 16802397

[3] C. Baleizão , H. Garcia , Chem. Rev 2006, 106, 3987. 16967927 10.1021/cr050973n

[4] K. D. M. Harris , S. J. M. Thomas , ChemCatChem 2009, 1, 223.

[5] B. Borca , T. Michnowicz , F. Aguilar‐Galindo , R. Pétuya , M. Pristl , V. Schendel , I. Pentegov , U. Kraft , H. Klauk , P. Wahl , A. Arnau , U. Schlickum , J. Phys. Chem. Lett. 2023, 14, 2072. 36799542 10.1021/acs.jpclett.2c03575 PMC9986952

[6] K. Mishra , D. Guyon , J. S. Martin , Y. Yan , J. Am. Chem. Soc. 2023, 145, 17242. 37499231 10.1021/jacs.3c04593 PMC10926773

[7] J. Ravi , D. Venkatakrishnarao , C. Sahoo , S. R. G. Naraharisetty , N. Mitetelo , A. A. Ezhov , E. Mamonov , T. Murzina , R. Chandrasekar , ChemNanoMat 2018, 4, 764.

[8] N. Mitetelo , D. Venkatakrishnarao , J. Ravi , M. Popov , E. Mamonov , T. V. Murzina , R. Chandrasekar , Adv. Opt. Mater. 2019, 7, 1801775.

[9] S. Li , B. Lu , X. Fang , D. Yan , Angew. Chem., Int. Ed. 2020, 59, 22623. 10.1002/anie.202009714 32875702

[10] J. A. Switzer , H. M. Kothari , P. Poizot , S. Nakanishi , E. W. Bohannan , Nature 2003, 425, 490. 14523441 10.1038/nature01990

[11] B.‐C. Iacob , E. Bodoki , A. Florea , A. E. Bodoki , R. Oprean , Anal. Chem. 2015, 87, 2755. 25630982 10.1021/ac504036m

[12] X. Mao , H. Zhao , L. Luo , D. Tian , H. Li , J. Mater. Chem. C 2015, 3, 1325.

[13] C. C. Negut , R.‐I. S.‐V. Staden , J. Electrochem. Soc. 2021, 168, 067517.

[14] J. Li , M. Telychko , J. Yin , Y. Zhu , G. Li , S. Song , H. Yang , J. Li , J. Wu , J. Lu , X. Wang , J. Am. Chem. Soc. 2021, 143, 10177. 34227379 10.1021/jacs.1c03091

[15] J. Sotres , H. Boyd , J. F. Gonzalez‐Martinez , Nanoscale 2021, 13, 9193. 33885692 10.1039/d1nr01109j

[16] Z. Zhu , J. Lu , F. Zheng , C. Chen , Y. Lv , H. Jiang , Y. Yan , A. Narita , K. Müllen , X.‐Y. Wang , Q. Sun , Angew. Chem., Int. Ed. 2022, 61, 202213503. 10.1002/anie.202213503 36178779

[17] M. Rashidi , R. A. Wolkow , ACS Nano 2018, 12, 5185. 29790333 10.1021/acsnano.8b02208

[18] S. V. Kalinin , M. Ziatdinov , J. Hinkle , S. Jesse , A. Ghosh , K. P. Kelley , A. R. Lupini , B. G. Sumpter , R. K. Vasudevan , ACS Nano 2021, 15, 12604. 34269558 10.1021/acsnano.1c02104

[19] A. Krull , P. Hirsch , C. Rother , A. Schiffrin , C. Krull , Commun. Phys. 2020, 3, 1.

[20] S.‐H. Yang , W. Choi , B. W. Cho , F. O.‐T. Agyapong‐Fordjour , S. Park , S. J. Yun , H.‐J. Kim , Y.‐K. Han , Y. H. Lee , K. K. Kim , Y.‐M. Kim , Adv. Sci. (Weinheim, Ger.) 2021, 8, 2101099. 10.1002/advs.202101099 PMC8373156 34081415

[21] B. Möller , J. Pirklbauer , M. Klingner , P. Kasten , M. Etzkorn , T. J. Seifert , U. Schlickum , T. Fingscheidt , in Proceedings of the IEEE/CVF Conference on Computer Vision and Pattern Recognition (CVPR) Workshops , Vancouver, BC, Canada, Jun 2023, 4263.

[22] Y. Liu , Q. Sun , W. Lu , H. Wang , Y. Sun , Z. Wang , X. Lu , K. Zeng , Adv. Theory Simul. 2019, 2, 1800137.

[23] M. Ziatdinov , A. Ghosh , C. Y. Wong , S. V. Kalinin , Nat. Mach. Intell. 2022, 4, 1101.

[24] S. Somnath , C. R. Smith , N. Laanait , R. K. Vasudevan , A. Ievlev , A. Belianinov , A. R. Lupini , M. Shankar , S. V. Kalinin , S. Jesse , (Preprint) arXiv:1903.09515, v2, submitted: Mar 2019.

[25] C. Draxl , M. Scheffler , JPhys Mater. 2019, 2, 036001.

[26] M. Scheffler , M. Aeschlimann , M. Albrecht , T. Bereau , H.‐J. Bungartz , C. Felser , M. Greiner , A. Groß , C. T. Koch , K. Kremer , W. E. Nagel , M. Scheidgen , C. Wöll , C. Draxl , Nature 2022, 604, 635. 35478233 10.1038/s41586-022-04501-x

[27] K. Choudhary , K. F. Garrity , A. C. E. Reid , B. DeCost , A. J. Biacchi , A. R. H. Walker , Z. Trautt , J. Hattrick‐Simpers , A. G. Kusne , A. Centrone , A. Davydov , J. Jiang , R. Pachter , G. Cheon , E. Reed , A. Agrawal , X. Qian , V. Sharma , H. Zhuang , S. V. Kalinin , B. G. Sumpter , G. Pilania , P. Acar , S. Mandal , K. Haule , D. Vanderbilt , K. Rabe , F. Tavazza , npj Comput. Mater. 2020, 6, 173.

[28] E. Rabani , D. R. Reichman , P. L. Geissler , L. E. Brus , Nature 2003, 426, 271. 14628047 10.1038/nature02087

[29] C. Wang , H. Li , Z. Hao , X. Li , C. Zou , P. Cai , Y. Wang , Y.‐Z. You , H. Zhai , Chin. Phys. B 2020, 29, 116805.

[30] U. Schlickum , R. Decker , F. Klappenberger , G. Zoppellaro , S. Klyatskaya , W. Auwärter , S. Neppl , K. Kern , H. Brune , M. Ruben , J. V. Barth , J. Am. Chem. Soc. 2008, 130, 11778. 18693686 10.1021/ja8028119

[31] D. Kühne , F. Klappenberger , R. Decker , U. Schlickum , H. Brune , S. Klyatskaya , M. Ruben , J. V. Barth , J. Phys. Chem. C 2009, 113, 17851. 10.1021/ja809946z 19256496

[32] T. Wang , Q. Fan , L. Feng , Z. Tao , J. Huang , H. Ju , Q. Xu , S. Hu , J. Zhu , ChemPhysChem 2017, 18, 3329. 28910515 10.1002/cphc.201700769

[33] F. Klappenberger , D. Kühne , W. Krenner , I. Silanes , A. Arnau , F. J. G. D. Abajo , S. Klyatskaya , M. Ruben , J. V. Barth , Nano Lett. 2009, 9, 3509. 19534501 10.1021/nl901700b

[34] S. Klyatskaya , F. Klappenberger , U. Schlickum , D. Kühne , M. Marschall , J. Reichert , R. Decker , W. Krenner , G. Zoppellaro , H. Brune , J. V. Barth , M. Ruben , Adv. Funct. Mater. 2011, 21, 1230.

[35] M. Pivetta , G. E. Pacchioni , E. Fernandes , H. Brune , J. Chem. Phys. 2015, 142, 101928. 25770517 10.1063/1.4909518

[36] B. D. Baker Cortés , M. Stöhr , in Encyclopedia of Interfacial Chemistry (Eds. K. Wandelt ), Elsevier, Oxford, 2018.

[37] G. Jocher , A. Chaurasia , A. Stoken , J. Borovec , NanoCode012 , Y. Kwon , K. Michael , TaoXie , J. Fang , imyhxy , Lorna , Z. Yifu , C. Wong , A. V. D. Montes , Z. Wang , C. Fati , J. Nadar , Laughing , UnglvKitDe , V. Sonck , tkianai , yxNONG , P. Skalski , A. Hogan , D. Nair , M. Strobel , M. Jain , (Preprint) zenodo:7347926, submitted: Nov 2022.

[38] jiali1025 , zhuyixintc , Long1Corn , J. Am. Chem. Soc. 2021, 143, 10177.34227379

[39] R. Padilla , S. L. Netto , E. A. B. da Silva , in,2020 International Conference on Systems, Signals and Image Processing (IWSSIP), Niteroi, Brazil, 2020, p. 237.

