# Supplementary material for: Chirality Detection in Scanning Tunneling Microscopy Data Using Artificial Intelligence
Source: Small Methods. 2024 Sep 9;8(12):2400549. doi: 10.1002/smtd.202400549 (PMC11671859; doi:10.1002/smtd.202400549)
Supplement: Supplementary file 1 — Supporting Information [file SMTD-8-2400549-s001.pdf]

small methods

Supporting Information

for *Small Methods*, DOI 10.1002/smtd.202400549

Chirality Detection in Scanning Tunneling Microscopy Data Using Artificial Intelligence

Tim J. Seifert, Mandy Stritzke, Peer Kasten, Björn Möller, Tim Fingscheidt, Markus Etzkorn, Timo de Wolff and Uta Schlickum**

Chirality Detection in Scanning Tunneling Microscopy Data Using Artificial Intelligence

Tim J. Seifert Mandy Stritzke Peer Kasten Björn Möller Tim Fingscheidt Markus Etzkorn Timo de Wolff Uta Schlickum**

S1 Basics of machine learning

A neural network is a type of machine learning model that is composed of a number of interconnected nodes that process information. The strengths of these connections are called *weights*. The networks are trained to perform a specific task like regression, image classification, or object detection. In our case, for the training, images of a *training dataset* are fed into the network, which processes the information contained in the pixel values in a black-box fashion and outputs a prediction. In our case, the prediction consists of a set of bounding boxes, each enclosing an object, and the corresponding object class. This output is compared to the true *label* (*ground truth*), which describes the correct output for the given input. The difference between the model output and true labels yields the error of the network. Using this error, the model optimizes its weights aiming to reduce the error once it is faced with the same or similar images.
A pass through all images in the training set is called an *epoch*. Ideally, the model's error decreases with the number of epochs. However, if the model would simply memorize every image contained in the training set, then it would, despite a small loss value, with a high probability, not perform well on a slightly different image set. To avoid this *overfitting* behavior, we track the error of a hold-out dataset, called *validation set*, during the training time. Initially, both the error on the training set as well as on the validation set decreases with the number of epochs, since the model learns how to output the correct solution. If the model is overfitted, then the error on the validation set starts to increase. This point is a good indicator to terminate the training process to obtain a model that actually learned the underlying concept of the task, and thus has a high chance to also achieve a high performance on unseen, new images. A set of such new images is called *test dataset*; it is used to perform the final model evaluation and comparisons between different models.
In an optimal situation, the training, validation, and test set belong to the same global dataset, such that we observe the same performance on all three datasets. However, in many real-world applications, this is not achievable. In particular, if there are not sufficiently many labeled real images available. In this case we switch to synthetic datasets for training and we expect to see a performance gap between the evaluation on the synthetic training and validation data versus evaluation on the real test data. To obtain a robust model with respect to changing from synthetic to real data, we can apply *augmentation* to the training data, increasing both its size and variety. In the case of image data, augmentation can be a modification of orientation, contrast, or saturation of the images.
A model consists of various processing layers, starting with an *input layer*, an arbitrary number of *hidden layers*, and the final *output layer*. Each layer consists of a finite number of nodes. Usually, two successive layers are connected to each other, i.e., the subsequent layer can access the information output by its predecessor, and in this way the information contained in the input is passed through the entire network. Using the weights of the involved nodes and *activation functions* the information is changed when passing from one layer to its successor. The information passing through the output layer is the prediction of the network. Hence, changing the weights of the nodes leads to a change of the model output. Depending on the task to be solved, different types of layers are combined. In object detection, *convolutional layers* extract the information contained in different areas in the input image and combine them to reasonable predictions. The term *model architecture* describes the specific combination of layers. Neural networks containing convolutional layers are called *convolutional neural networks (CNN)*. In the case of object detection, the output of such a model is a set of four bounding box coordinates, a prediction of which class the enclosed object belongs to, and a *confidence score* indicating how certain this prediction is.

S2 *Aug* Training Data

Li et al. choose the image, upon which the training data generation will be based, according to a set of criteria. Importantly, the objects of different chirality should have a high separation in the t-SNE visualization. T-SNE is often used for data exploration and visualization of high dimensional data.[1] In this presented work, each object in an STM-image is manually cropped, and scaled to the same size. The resulting image then represents one data point, whose features are the pixel values. Thus, the dimension of the data space is the number of pixels. With t-SNE, the dimension is reduced to only two features. Visualizing these features in a scatter plot leads to a graphical representation where two points are, roughly speaking, located close to each other with high probability, if their preimages had a small Euclidean distance (compared to the other data points). Hence, if two objects are close in the new representation, it means that they resemble each other in terms of textures and color gradients. It is important to note that this method captures only local patterns extracted from the pixel values and does not provide a global understanding. We refer to the original work cited above for an in depth explanation. Our main takeaway is: If the data points of each class exhibit suitable clustering patterns in this reduced form, then the image is probably more suitable as an input for the creation of the dataset.

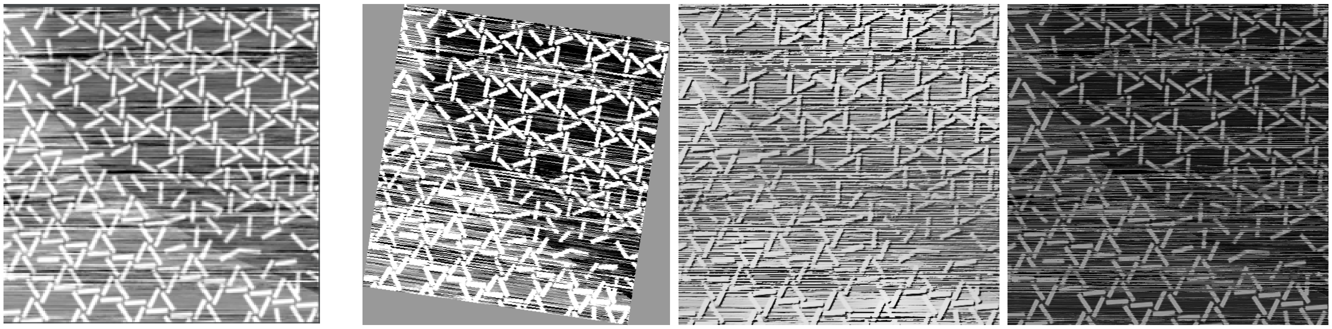

(a) (b)

Figure S1: Dataset *Aug* is created from the initial image (a) with the method by *Li et al.* Sample images are shown in (b)

S3 Synthetic training Data

The synthetic dataset *Synth.* used to train the various stages has been generated by a custom, highly-customizable python script. After the structure is defined once, the data generation and labeling is fully automated and provides a very variable dataset, as our investigations have shown.

S3.1 Concept and parameters

Simulating highly accurate STM images is used very commonly using mostly Density Functional Theory (DFT) methods.[2] The downside of this physical accuracy is an extremely high computational cost, at a cubical scaling with the number of atoms in the simulation.[3] This makes generating datasets for the training of machine learning algorithms technically impossible within reasonable timescales. In this work, a much simpler, not quantum-mechanically accurate method as employed, which comes with the upside that thousands of images can be generated within few hours.
In concept, the molecular appearance, as well as its alignment behavior are imported in the program and modified using a variety of parameters to increase the flexibility of the dataset. These parameters, which are randomly sampled for each image, include

- The path of atomic steps within the image
- The angle between the lattice vector and the image axis
- The exact position and angle of each molecule
- The apparent physical size of the image, quantified as number of pixels per unit cell
- The apparent height of the molecules and atomic steps
- The sharpness of the molecules (see Section S3.4)
- The chirality of each domain
- The number of structural defects in the image (see Section S3.4)
- The slope within the image (see Section S3.4)
- The mean and variance of each noise type (see Section S3.5)

The image generation process works as follows:

S3.2 Structural definition

The shape of each molecule follows a simple function mapping relative 2D coordinates onto a height. In this case, a simple rectangular model was sufficient, whose lengths, width and height have been determined from a manual measurement of real STM data, but a single (DFT-) simulated image, or just the chemical model would have been possible as well.
Apart from the appearance of each molecule, the molecular arrangement needed to be predefined as well. For this, the molecular unit-cell, as well as the lattice vectors had to be defined. As for the individual molecules, this is simply possible by measuring real data, or by theoretical calculations. Multiple possible alignments can be used just as well, as here utilized to define different chiralities.
For the kagomé lattice, the hexagonal unit-cell was chosen and defined separately for each chirality. For the $NC{-}Ph_4{-}CN$ structure, the primitive unit-cell consists of just two molecules, each also modeled as rectangles, just with shorter length compared to $NC{-}Ph_5{-}CN$.
After this setup, the following steps were automated according to the defined parameters, generating datasets of vast size automatically.

S3.3 Molecular alignment

The physical size of each image is defined by the pixels of the image, as well as its resolution in nanometer (edge length) per pixel. This is used to translate measurements in pixels (for visualization or labeling) to physical quantities (lattice vectors or molecular dimensions).
The molecular arrangement has been simulated by first randomly choosing a starting point within the image region. The lattice vectors were rotated with a random angle to virtually rotate the entire molecular network. From this starting point on, the entire image area was patterned with unit cells according to the lattice vectors.
With a certain probability, the image should contain an atomic step. The path of the atomic step was modeled as a random walk with drift. A starting point for this step was randomly sampled at one edge of the image. The path was sampled in discrete steps: Assuming an atomic step from left to right and starting from an initial velocity v_0 and y-coordinate y_0, the path would be sampled as

$$v_x \leftarrow v_{x-1} + \mathcal{U}(-\lambda, \lambda) \tag{1}$$

$$y_x \leftarrow y_{x-1} + v_x \tag{2}$$

with a uniform distribution $\mathcal{U}$ centered around zero with a parameter λ defining the rate of change in this curve. For computational speedup, the curve was interpolated between larger steps.
If there was an atomic step present, molecules were aligned at both domains separately with different lattice angles and potentially different chiralities. Nearby the step, molecules were removed with a linearly increasing probability approaching one at a molecule length away from the slope. Additional randomly aligned molecules nearby the step edge were added. Visually, the atomic step was recreated as a sigmoid function, resulting in a higher and a lower side with a smooth transition.

S3.4 Structural and Instrumental Artifacts

After the alignment was simulated, several steps were taken to increase the realism of this simple model. First, instead of using the rectangular model for the molecules, the shape was altered to resemble tip-convolution effects. The height profile $h(x)$ across each side of the rectangle was switched to the smoother sigmoid function

$$h(x) = \frac{1}{\exp(\gamma \cdot x) + 1} \tag{3}$$

with a smoothing parameter γ chosen randomly for each image.
Structural impurities were added and visualized as a series of partially overlapping circles. Finally, since experimentally the STM sample is never perfectly perpendicular to the tip, a slope was included by adding a linearly position dependent height to the image.

S3.5 Noise disturbances

Even more prominent in STM images is the noise, which was modeled as a combination of general white noise, as well as scan-induced line-wise noise. The white noise contribution was included by adding height values sampled from a normal distribution to each pixel in the image, where the mean and standard deviation were randomly defined for each image.
The line-wise scanning noise was modeled as noise with a $1/f^{\beta}$ power dependency. This was individually sampled per scan-line, as well as for each line in the image, again with predefined standard-deviation. The implementation is based on the work of *Kasdin*.[4]

S3.6 Data labeling

Apart from the images themselves, labels are necessary to teach the ML algorithm what to look for. In this case, object detection was used which means that box coordinates are needed for each object in the image. Due to the rotational symmetry of this structure, oriented bounding boxes were not used, hence

the bounding boxes were always aligned with the image axes. The boxes shape was defined as the smallest possible box to contain all molecules assigned to this unit-cell. Since the molecules are not perfectly sharp, the outline of each individual molecule was chosen as the point, at which the sigmoid function (eq. 3) drops to 10 %. If the unit-cell was not complete, e.g. at an atomic step, a threshold of at least 3 molecules that need to be present was chosen. Also at the edge of the image, at least 30 % of the total unit-cells area needed to be visible in the image. Since in the kagomé structure the position, height profile, chirality, and affiliation with unit-cells is known by design, the calculation of the bounding box coordinates was straightforward. For the NC−Ph_4−CN structure, the object to identify was defined as four molecules forming an open square, but the primitive unit-cell only consisted of two molecules. Therefore this required a little more effort to calculate the affiliation with both objects for each molecule.
For additional information, the code is provided at `https://moto.math.nat.tu-bs.de/appliedalgebra_public/chirality_detection_in_stm_via_artificial_intelligence.git`.

S4 Technical details for ML architecture setups

To improve the results obtained by model F, especially when training on dataset *Synth*, multiple parameters in the setup of our Faster R-CNN architecture wre changed to better adapt to the given task and training set. These changes are listed in Table S1:

Parameter	original publication	our adaption
INPUT.MIN-SIZE-TRAIN	(50, 450)	(510, 520)
INPUT.RANDOM-FLIP	horizontal	none
MODEL.ROI-HEADS.BATCH-SIZE-PER-IMAGE	512	750
MODEL.RPN.BATCH-SIZE-PER-IMAGE	256	750
MODEL.ANCHOR-GENERATOR.SIZES	[16,32,64,128]	[4,8,16,32,64,128]
MODEL.RPN.POSITIVE-FRACTION	0.5	0.8
TEST.DETECTIONS-PER-IMAGE	100	2000

Table S1: Changed parameters from model F to F^*

The incorporation of two smaller anchors was very important. As a two stage detector, Faster R-CNN does not predict objects directly in a unified network, but instead uses a separate Region Proposal Network (RPN) to generate boxes which might contain an object and provides these candidates to the detection stage. The RPN uses anchors as initial guesses, or reference points for its detections. Apart from different aspect ratios, anchors also have different sizes, which are defined in the setup of the architecture. If there are no anchors matching the size of an object in the image, its recognition poses a significantly harder task. This can be crucial for the detection capabilities, especially for extreme box sizes as observed in our experiments. Therefore, anchors of size 4 and 8 were included to the list of anchor sizes, set as MODEL.ANCHOR–GENERATOR.SIZES. Furthermore, since flipping the image changes the chirality, random flipping was disabled.

For the YOLO setup, mostly the default parameters were used. However, since large-scale images may contain a large number of unit cells, the number of maximum detections was increased to 5 000. As before, the flipping procedures for augmentation were disabled.

S5 Metrics for Object Detection

To compare our models on a quantitative basis, we use well-established metrics, and then we introduce some modifications for our specific setup. For a more detailed overview, we refer, e.g., to *Padilla et al.* [5]

Precision, Recall: For the evaluation of a single image, *precision* and *recall* scores were calculated. A prediction of a model was considered as *correct*, if the *intersection over union* (IoU), given as the intersection area of two boxes divided by their union area, with the ground truth box was above a certain threshold and the predicted class matched the ground truth label. The *precision score* was determined by dividing the number of correct predictions by the total number of predictions made by the model. Thus, this score describes how often the model is correct when it outputs a certain object. In contrast, the *recall score* captures the ratio of the number of correctly detected objects in the prediction to the total number of objects in the ground truth. Both precision and recall depend on the IoU threshold. In our experiments, this threshold was set to 50%, i.e., a box output by a model is only considered as correct if its IoU with the ground truth box is at least 50%. This choice was motivated by the present dataset: Since the objects contained in the images were very small at larger image sizes, shifting a predicted box by a few pixels could mathematically result in a much lower IoU, while the prediction was perceived as unaltered in the eyes of an observer.

Mean Average Precision: As mentioned in Section S1, the object detection models furthermore yield a confidence score. Both precision and recall scores were evaluated for different confidence thresholds over the entire dataset. The *precision-recall curve* was obtained by depicting the results as interpolated data points in a two-dimensional coordinate system. The area under this curves is defined as *mean average precision* (mAP), which is a widely used metric in object detection. It takes values between 0 and 1, where a higher mAP indicates a better performance. One constraint for this metric, however, is its sensitivity at small object sizes, since the IoU of two boxes is very susceptible to small translations at small box sizes.[6] The performance was analyzed in more detail by creating 21 intervals over the images sizes and calculating their respective mAP. In this way, an improved perception of the performance with respect to different images sizes was obtained. Starting at $\text{border}_{\text{left}} = 8\,\text{nm}$, the sequence of interval borders was recursively set as $\text{border}_{\text{right}} = \sqrt[4]{2} \cdot \text{border}_{\text{left}}$. The last interval was cut off at 300 nm. This interval design was chosen since the overall image appearance changes more in the lower image sizes than in the larger ones, i.e., the visual difference between 24 nm and 30 nm is larger than between 124 nm and 130 nm. Recall that the mAP is calculated over the total number of bounding boxes present in the whole dataset (and not as a mean of the precision over all images in the dataset).

Average mAP ⟨mAP⟩: Since the aim was to train a *„One fits most image sizes“* model, the ⟨mAP⟩ was calculated additionally as an average over all mAP per size interval. In doing so, a metric was constructed, that reflects the performance at different image sizes better than the mAP alone, summarized in one single value. However, this is only beneficial if the distribution of images per size interval is representative for the *„One fits most“*-approach. If the number of samples is highly imbalanced, the ⟨mAP⟩ can be strongly biased towards intervals which contain a lower number of images. Thus, this ⟨mAP⟩ was calculated only on synthetic images, where a balanced distribution is guaranteed. For measuring the performance on real images,the regular mAP was used.

S6 Precision, Recall and Confidence

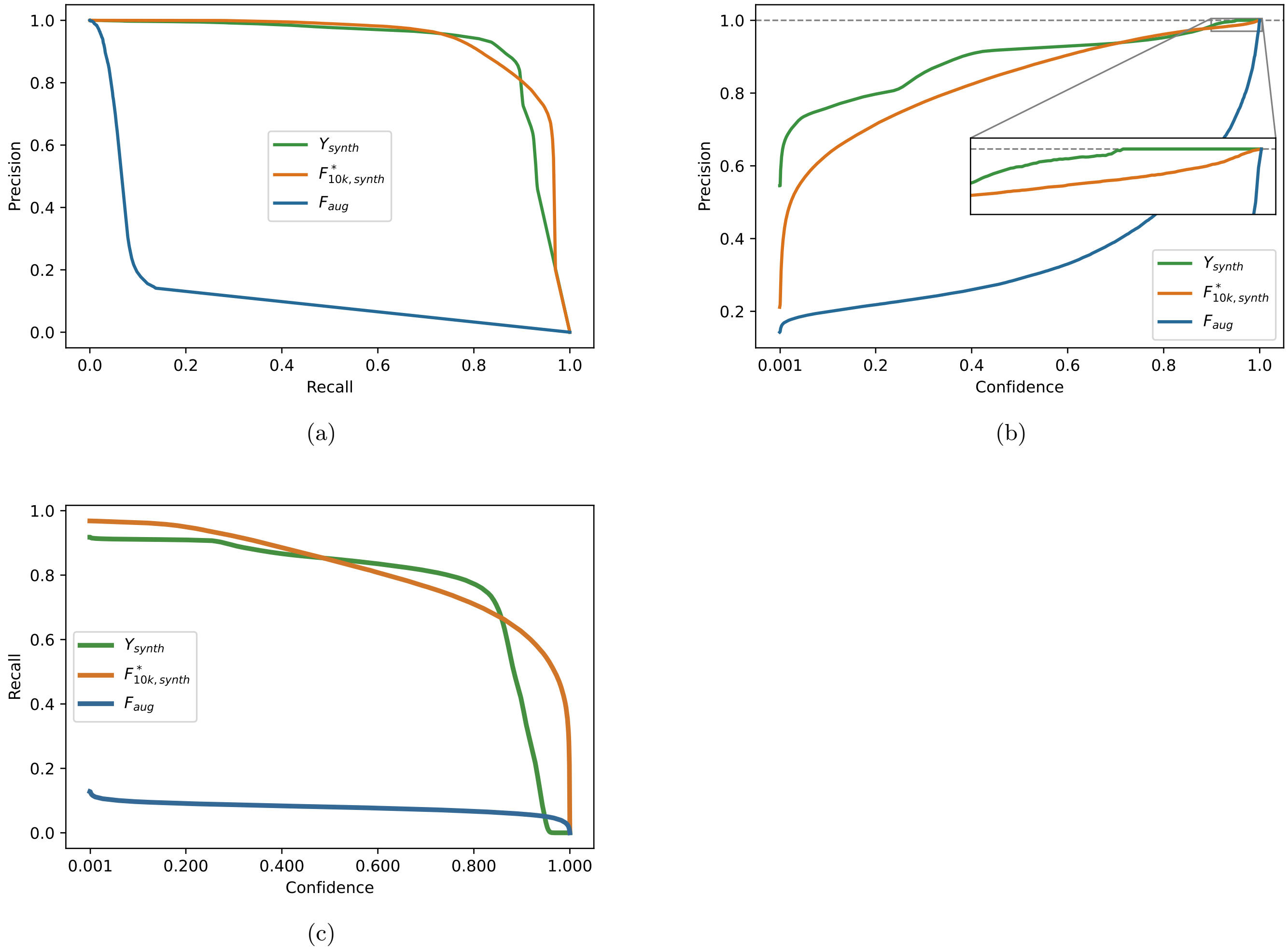

Figure S2: Comparison of the Precision-Recall curve (a), Precision-Confidence curve (b) and Recall-Confidence curve (c) for models F^*_{10k} and Y, both trained and evaluated on the synthetic dataset *Synth*, as well as for model F_{aug} trained on the augmented dataset.

The relevant differences between model F^*_{10k} and the YOLO architecture are presented in Figure S2. In Figure S2a, the precision-recall curve for both models is observed. Additionally, the curve for model F_{aug} is shown as a reference.

Upon initial observation, the curves for scenarios $F^*_{10k,Synth}$ and Y_{Synth} exhibit similar behavior. While this holds true at equally excellent precision for a recall up to 0.7, the curves differ in the high-recall regime: As seen in Figure S2a, two crossing points are observed, resulting in recall ranges in which YOLO or Faster R-CNN perform better, the latter succeeding even at very high recall.

In Figure S2b, this behavior was analyzed in more depth, showing precision over confidence. Overall, YOLO manifests a higher precision value for a given confidence than Faster R-CNN. Especially for very high confidences, YOLO surpasses Faster R-CNN and produces predictions with a very high precision. Faster R-CNN on the other hand can be beneficial in scenarios where recall rates close to one are required, since it maintains higher precision then YOLO as can be seen in Figure S2a.

As becomes apparent in both figures, model F_{aug} displays a significantly different behavior, again underlining that this model does not succeed in the kagomé detection.

Additionally, Figure S2c shows the recall over the confidence. While F_{aug} stays at a low recall independent of the confidence threshold, $F^*_{10k,Synth}$ and Y_{Synth} show high recall. Model $F^*_{10k,Synth}$ shows an initially higher recall, which declines gradually over a long range, whereas the recall for model Y_{Synth} drops abruptly, showing better recall than Faster R-CNN only for confidence thresholds between 0.5 and 0.85.

We want to point out, that when two models show very similar mAP performance for a certain image size, this does not mean that the predictions are alike for each individual image. Showing predictions at a certain confidence threshold is can behave differently for different models, as also indicated in the Precision-Confidence and Recall-Confidence curves. This is especially the case for large-scale images, in which the number of pixels per unit cell is barely sufficient to resolve the internal chiral structure. Here the detection is very fragile with different models yielding vastly different outcomes for individual images, even though the models perform very much alike when averaged over multiple images.

S7 Inference times and mAP for different sizes

Table S2: mAP averaged over the image sizes intervals (= $\langle \text{mAP} \rangle$) for all eight models, evaluated on the synthetic Dataset *Synth*. Additionally, the $\langle \text{mAP} \rangle$ for images up to a size of 215 nm is calculated as well.

Stage	Inference time	Dataset *Synth*		Dataset *Aug*	
		$\langle \text{mAP} \rangle$	$\langle \text{mAP} \rangle_{\leq 215\text{nm}}$	$\langle \text{mAP} \rangle$	$\text{mAP}^*_{\leq 215\text{nm}}$
F	45.7 ms	0.562	0.616	0.237	0.262
F^*	254.9 ms	0.911	0.939	0.178	0.196
$F^*_{10\text{k}}$	252.1 ms	0.931	0.948	0.173	0.191
Y	81.3 ms	0.938	0.959	0.320	0.354

S8 Additional Faster R-CNN stages

S8.1 Setup

So far, four different model stages have been discussed:

Stage	Architecture	Dataset size	Epochs	Modifications
F	Faster R-CNN	1 000	16	Baseline settings by *Li et al.*
F^*	Faster R-CNN	1 000	16	Smaller anchors, no cropping (see S3)
F^*_{10k}	Faster R-CNN	10 000	1.6	Increase training set size
Y	YOLOv5	10 000	30	Usage of YOLO architecture (see S3)

The performance gains by increasing the dataset size and removing cropping procedures can be quantified separately by introducing three additional sub-stages. The construction of these stages is listed in Table S3 and visualized in Figure S3.

Table S3: Overview of the different models. Added models are highlighted.

Stage	Architecture	Dataset size	Epochs	Cropping	Anchors
F	Faster R-CNN	1 000	16	yes	default
F_{10k}	Faster R-CNN	10 000	1.6	yes	default
F^*_C	Faster R-CNN	1 000	16	yes	small
$F^*_{C,10k}$	Faster R-CNN	10 000	1.6	yes	small
F^*	Faster R-CNN	1 000	16	no	small
F^*_{10k}	Faster R-CNN	10 000	1.6	no	small
Y	YOLOv5	10 000	30	/	/

S8.2 Results

All models were trained on the augmented, as well as on the synthetic dataset. These models were evaluated on a synthetic test set. The results are listed in Section S8.2.

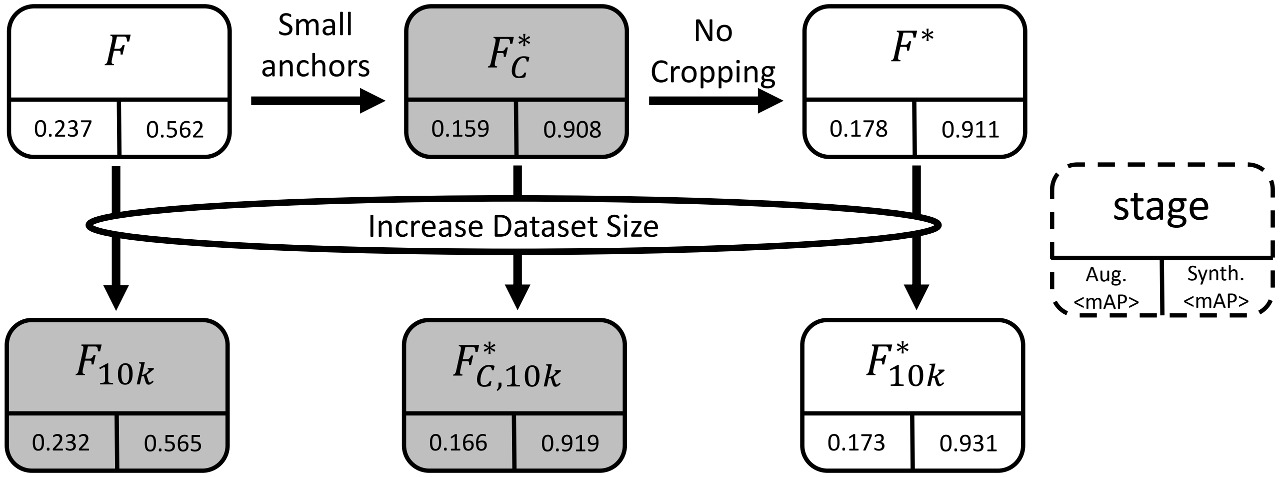

Figure S3: Evolution of the stages. White: previously used stages. Gray: additional stages for ablation study. ⟨mAP⟩ values from Table S4

Table S4: ⟨mAP⟩ for all setups and both training datasets, trained on either *Aug* or *Synth*, evaluated on the dataset *Synth*.

Stage	*Aug* training data	*Synth* training data
F	0.237	0.562
F_{10k}	0.232	0.565
F_C^*	0.159	0.908
$F_{C,10k}^*$	0.166	0.919
F^*	0.178	0.911
$F_{10\text{k}}^*$	0.173	0.931
Y	0.320	0.938

Throughout all experiments using augmented training data, it was observed that increasing the training set size changes the performance only marginally, increasing only for model F_C^* and decreasing for the others. When training on synthetic training data, an increase in performance with increased training set size was observed for all stages. We especially want to point out that the performance gain for stage F is negligible, increasing from 0.562 to 0.565. This once again shows that the model architecture itself strongly limits the achievable performance. For the other stages however, the benefit of a larger dataset is significant.

Additionally, by separating the removal of cropping procedures from the modification of anchor sizes, greatly different behaviors for training on synthetic and augmented training data are present. On augmented data, the introduction of smaller anchors weakens the performance from 0.237 (F) to 0.159 (F_C^*), which increased slightly to 0.178 (F^*) by removing cropping procedures.

Contrarily, using synthetic training data, the behavior is fundamentally different. The performance improvement almost exclusively stems from including smaller anchors, improving the ⟨mAP⟩ from 0.562 (F_{10k}) to 0.919 ($F_{C,10k}^*$). Removing cropping procedures for synthetic training data increased the performance only marginally, changing the ⟨mAP⟩ in transition from $F_{C,10k}^*$ to F_{10k}^* from 0.919 to 0.931.

S8.3 Discussion

The cropping procedures included in the baseline setup by *Li et al.* were intended as an additional augmentation procedure. The variety of data was increased, which came with the downside of reducing the image resolution. However, using synthetic training data, in fact a loss of performance due to cropping procedures was observed. This can be reasoned in the already very high diversity of the training data. Thus, the intended benefit of increasing the size variety in the data vanisheed, while the loss of image resolution takes effect and weakened the models performance. For the augmented data, a decrease in model performance when applying cropping was also witnessed. Since this dataset was created by applying augmentation, including cropping, the application of cropping inside the model once more did only lead to reduced image quality without providing additional variability.

We therefore conclude that especially the modified anchor sizes boost our performance significantly. Furthermore, the size of synthetic training data was broadly distributed, not requiring any cropping procedures in the augmentation.

S9 Results on high-zoom images

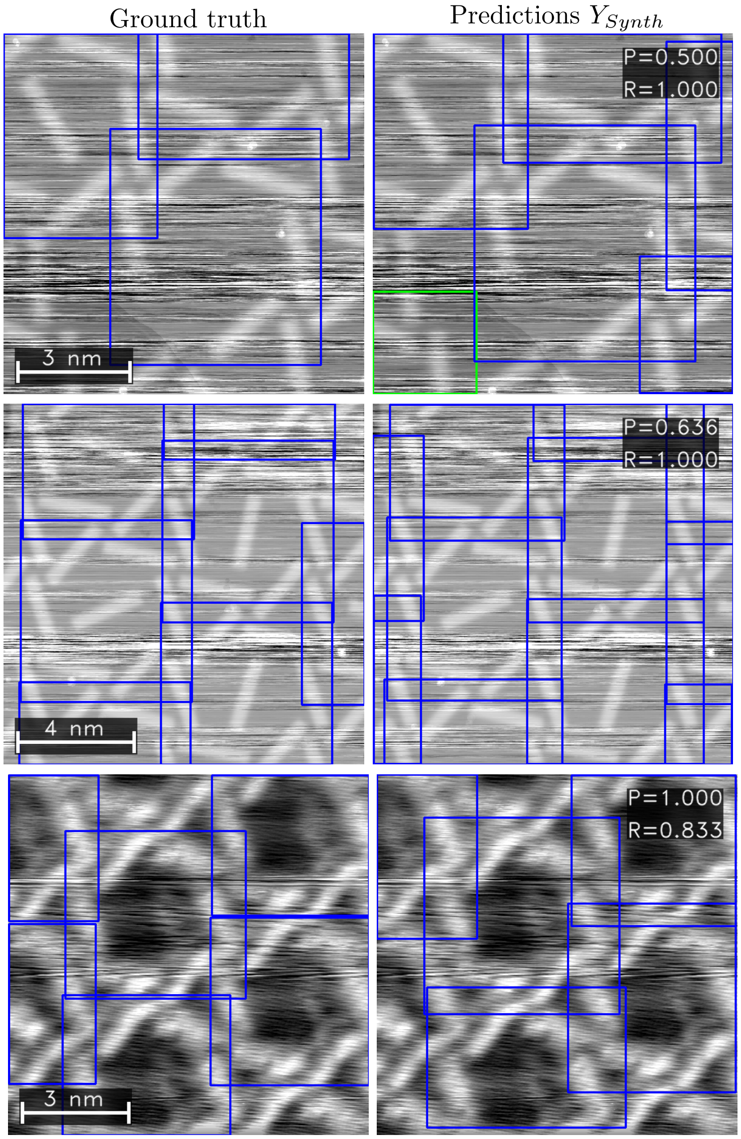

Figure S4: Example results using model Y_{Synth} on images with high zoom. At the edges of the images, objects were only partially displayed. Unit cells were included in the image if at least 30 % were visible. This causes missing or additional detections in some cases. These findings are equivalent for all well-performing models. First line: synthetic image of size 9.7 nm. Second line: synthetic image of size 12.3 nm Third line: real image of size 10.2 nm

S10 Visualization of different image sizes

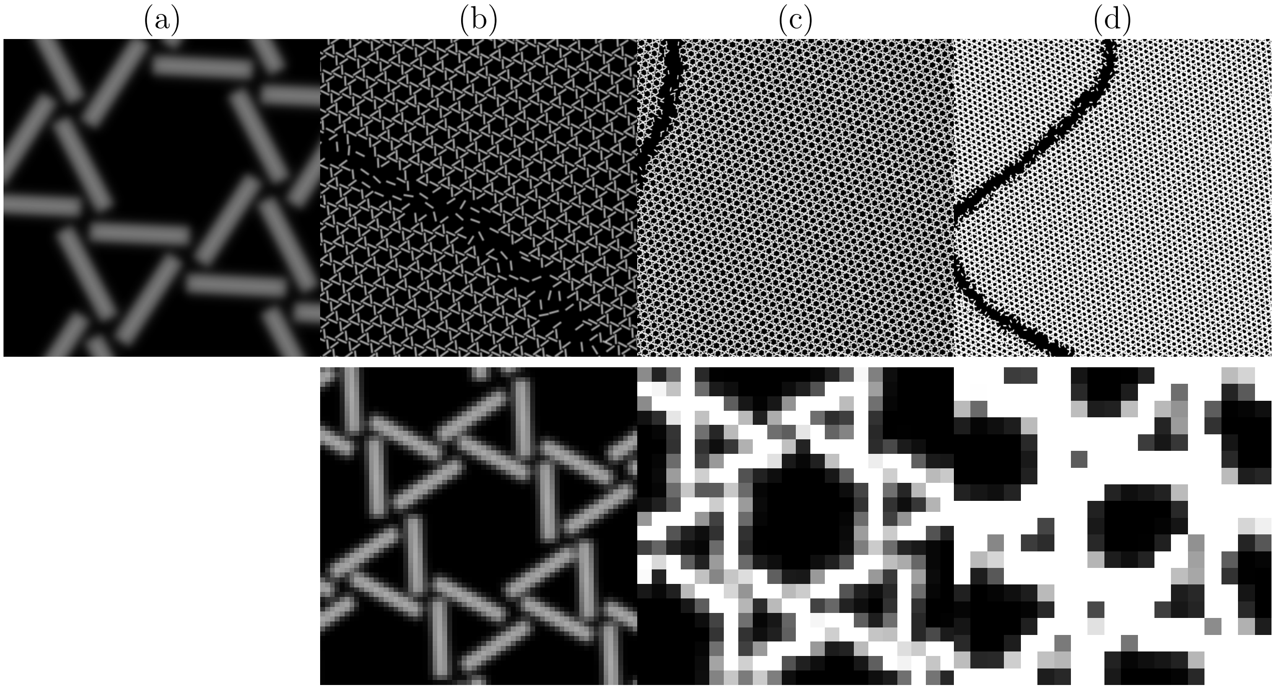

Figure S5: Synthetic images showing an range of 8 nm (a), 80 nm (b), 215 nm (c) and 300 nm (d) at 512 px resolution. Below are cut-outs of unit cells for each image.

S11 Comparison to different image resolution

Throughout this work, it has been claimed that the mAP results drop off at very high image sizes solely due to the limit in pixels per unit cell, and argued, that an increase in the image resolution will make detections for larger images possible. To test this, model $F^{*}_{\text{10k,synth}}$ was evaluated on synthetic data generated using the same methods as for dataset *Synth*, but increased image resolution by a factor of 1.5 to 768 px.

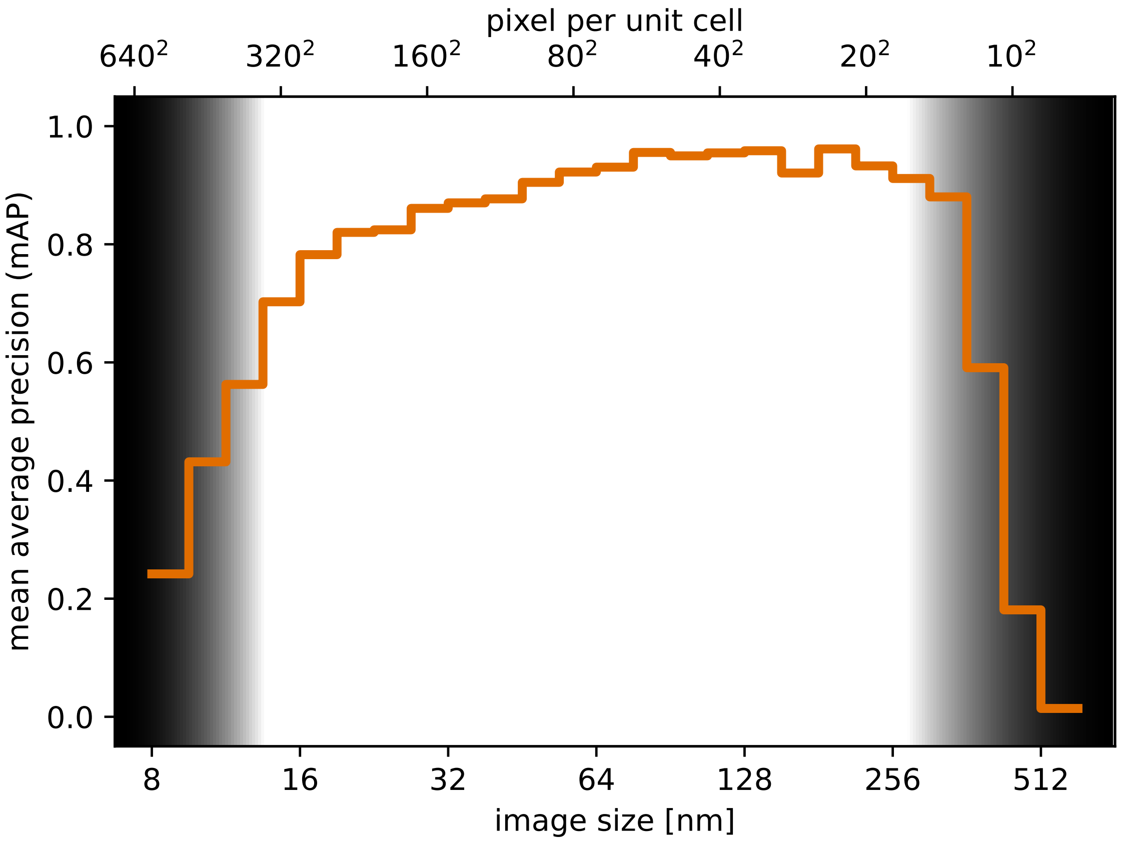

Figure S6: Evaluation of synthetic dataset with resolution increased to 768 px using model $F^{*}_{\text{10k,synth}}$. Within darker regions, manual investigation also gets increasingly difficult.

To compare, the mAP over the image size was plotted in Figure S6 and we conclude, that the point at which the performance drops off is shifted towards larger images, but still happens at approximately the same number of pixels per unit cell. Toward smaller images, the model now has a lower performance, since the very large unit cells with many pixels per unit cells were not part of the training set, since this was generated at a lower resolution of 512 px. The black bars indicate the areas, at which the evaluation is hindered by intrinsic reasons as already shown in the main paper in Figure 3. Toward small images, the boundary stays unaffected, since the issue of the small number and partly depicted unit cells is independent of the number of pixels. Toward large images, the boundary shifts outwards, since the issue of not enough pixels per unit cell occurs at larger image sizes due to the grater number of pixels in the image.

S12 Categorization of real images

For further evaluation, the real dataset was split according to their image size and quality. Here some exemplary images of each category are shown.

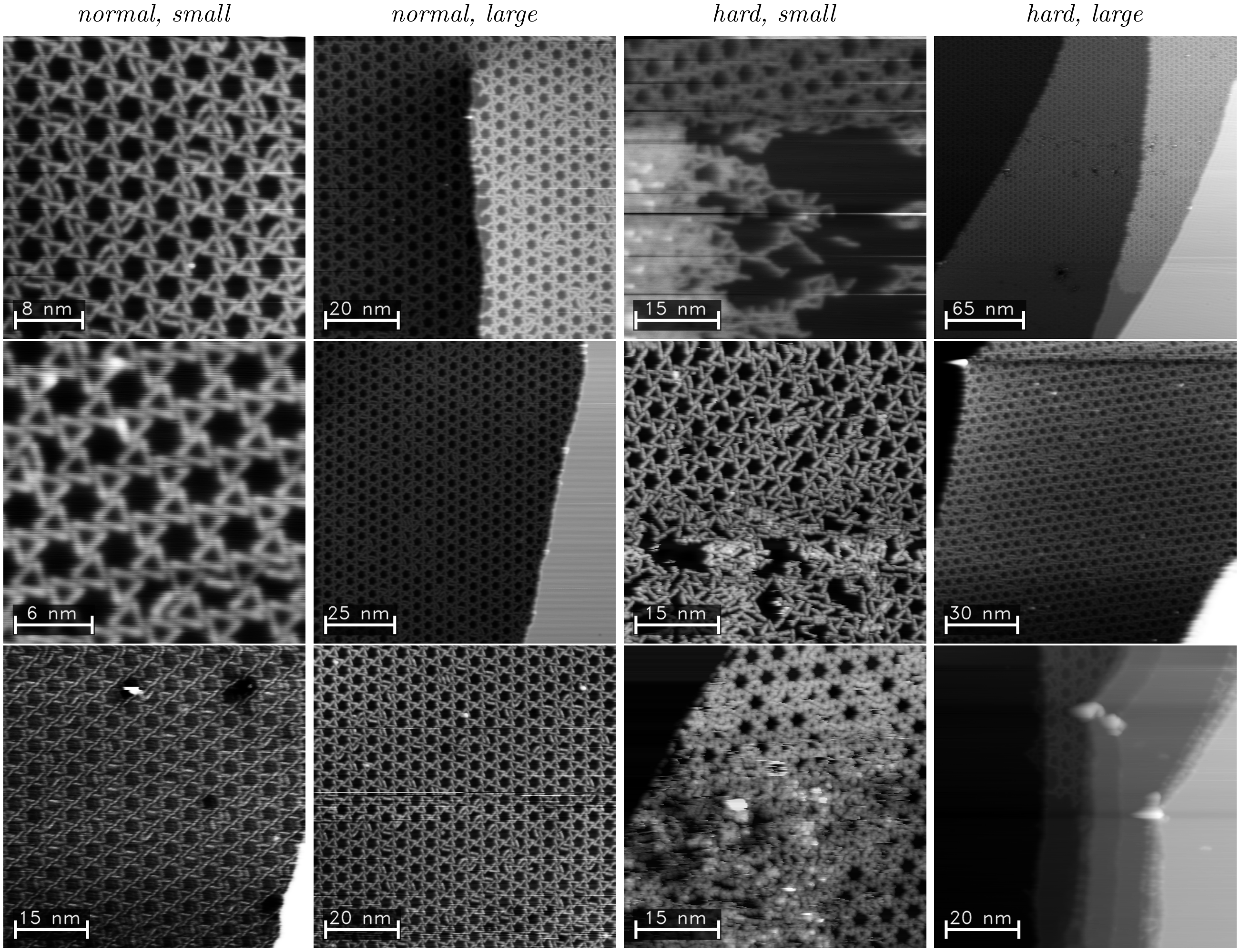

Figure S7: Example results for real images of different categories

S13 Results without preprocessing

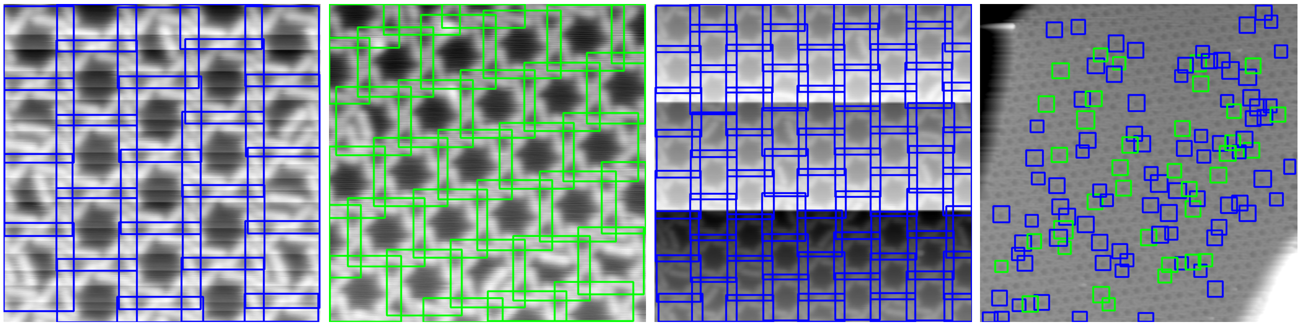

Figure S8: Example results for real images without preprocessing using model $F^{*}_{10k,\mathrm{synth}}$. On normal small images the model achieved an mAP of 0.99, this dropped to 0.66 for normal, but large images and to 0.60 for images labeled as *hard*.

S14 YOLOv5 training without early stopping

Table S5: mAP averaged over the image sizes intervals (=⟨mAP⟩) for all eight models, evaluated on the synthetic dataset *Synth*.

Stage	Inference time	Dataset *Synth*		Dataset *Aug.*	
		mAP*	$mAP^*_{\leq 215nm}$	mAP*	$mAP^*_{\leq 215nm}$
F	46 ms (45 ms)	0.562	0.616	0.237	0.262
F^*	256 ms (253 ms)	0.911	0.939	0.178	0.196
F^*_{10k}	253 ms (257 ms)	0.931	0.948	0.173	0.191
Y	81.7 ms (81.5ms)	0.938	0.959	0.32	0.354
Y-100	-	0.956	0.975	-	-

Table S6: Evaluation of the real images with the different models.

architecture	Dataset *Synth*			Dataset *Aug*		
	$mAP_{<80nm}$	$mAP_{\geq 80nm}$	mAP_{hard}	$mAP_{<80nm}$	$mAP_{\geq 80nm}$	mAP_{hard}
F	0.904	0.074	0.131	0.347	0.00029	0.064
F^*	0.988	0.934	0.694	0.552	0.004	0.043
F^*_{10k}	0.991	0.865	0.671	0.531	0.0025	0.037
Y	0.944	0.793	0.560	0.344	0.0045	0.035
$Y - 100$	0.969	0.815	0.596	-	-	-

By training the YOLO model on dataset *Synth* longer than the comparable Faster R-CNN networks, the performance can be increased even further, but just slightly. For synthetic data, this model now outperforms the different stages (Table S5), but on real data it cannot reach the performance of F^* and F^*_{10k} (Table S6). We conclude, that the marginal performance gain is not worth the longer training effort.

S15 YOLOv5 vs. YOLOv8

S15.1 Setup

The YOLO environment is still under heavy development with newer versions being released regularly. While the main investigations are based on the less recent, but well established and commonly used YOLOv5, probe potential advancements using YOLOv8 should now be probed as an example. YOLOv8 is the next and most recent major release. Apart from architectural and loss function improvements, one main difference to YOLOv5 is the step towards anchor-free detections, following a hybrid approach to reduce the dependency on the, however still present, anchor boxes. As the selection of anchors has shown to be crucial, YOLOv8 offers the potential to further enhance the detection performance.
The medium version of YOLOv8 has been trained on synthetic data on a *Nvidia GTX 1080 Ti* GPU. The first observation is the fact that the training of the newer YOLO version is slower. For YOLOv5, the number of epochs was chosen to match the training duration 2.5 h of the Faster R-CNN models, which resulted in 30 training epochs. For YOLOv8, in this time only 20 epochs could be completed, hence this serves as a reference. Additionally, we also compare the results after 30 epochs, as well as the best validating model after 100 training epochs for YOLOv8.

S15.2 Results

Table S7: Comparison of YOLO v5 vs. v8, trained on synthetic data.

Stage	synth	Real		
	⟨mAP⟩	$mAP_{<80nm}$	$mAP_{\geq 80nm}$	mAP_{hard}
YOLOv5	0.938	0.944	0.793	0.561
YOLOv8	0.979	0.972	0.444	0.361
YOLOv8 (30 Ep.)	0.980	0.988	0.923	0.641
YOLOv8 (Best Ep.)	0.987	0.984	0.924	0.607

The models were trained on synthetic data and evaluated on both, synthetic and real data. The results on real data are split between small, large, and hard images. The results are listed in Table S7.
For the reference model that was trained for the same time duration as our previous models, we indeed see an increase in ⟨mAP⟩ on synthetic test images from 0.938 up to 0.979, as well as on small real images (0.944 to 0.972). However, the performance is significantly worse on large and and hard real images, reaching only around 60 % of the previous results. Hence, this model is not as suitable for our purposes as the YOLOv5 version.
If, however, YOLOv8 is trained for the same number of epochs as the YOLOv5 version, greatly enhanced performance across all test cases, synthetic and real, was observed. Especially on large real images, the performance increased up to 0.923, compared to 0.793 for the older version. One reason for this improvement is the hybrid approach to anchor boxes, boosting the capabilities to detect small object appearing in large images. This has already been observed for the Faster R-CNN models, for which the incorporation of smaller anchors provided much better results in these cases.
Furthermore, even longer training helped to gain better performance only on the synthetic dataset, indicating that the model started to overfit to synthetic data and hence fell short in the transfer to real data.
In conclusion, YOLOv8 requires longer training than YOLOv5, hence with our set restrictions, its performance falls behind YOLOv5 and is therefore not used for in-depth analysis of all cases. However, if it is trained for longer, it surpasses YOLOv5 and becomes the best model for all test scenarios.

S15.3 mAP over image size

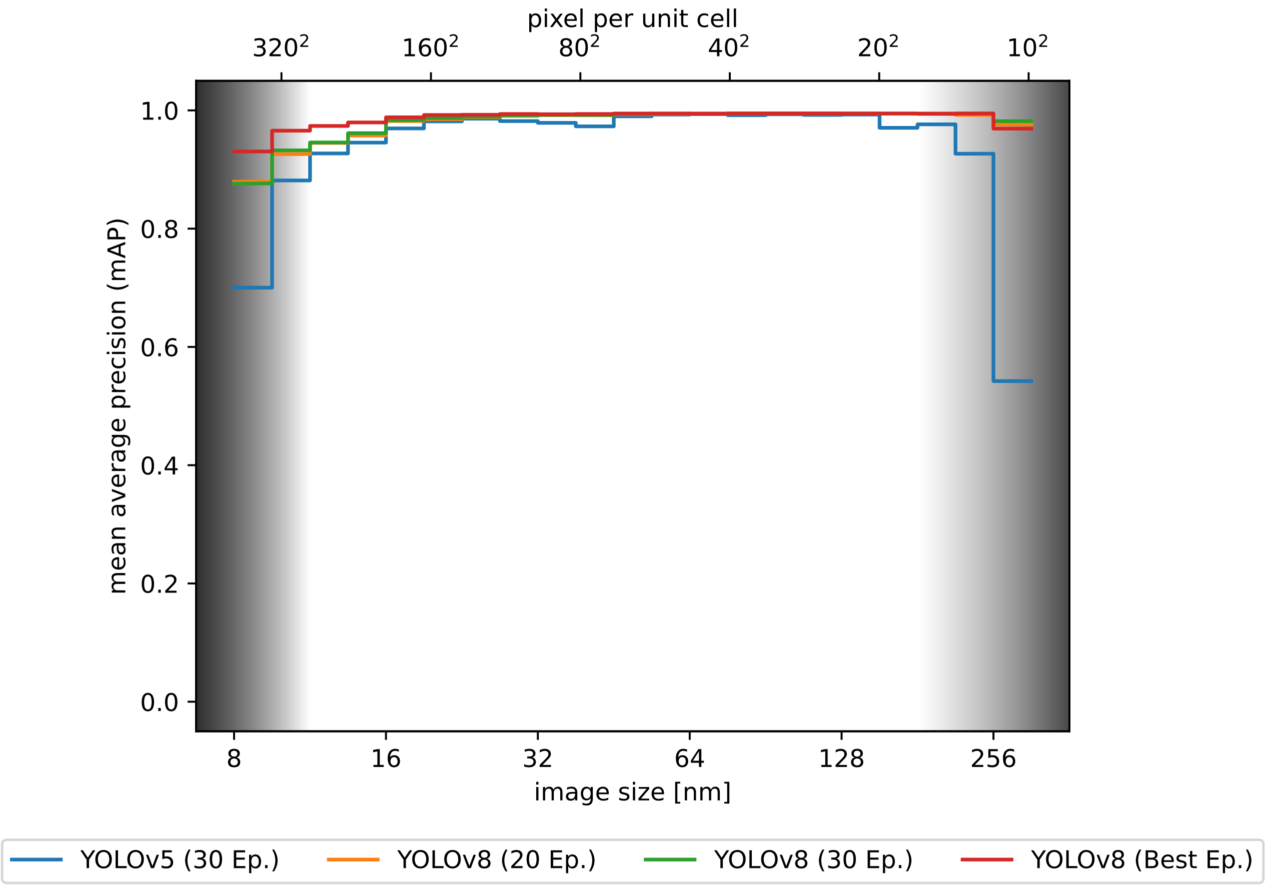

Figure S9: Mean average precision per image size for selected models evaluated on the dataset *Synth*. Gray regions indicate the area in which constraints of the experimental setup become important, limiting the achievable performance independently of the models themselves.

Similarly to the Faster R-CNN stages, the performance of the discussed YOLO models is compared in dependence on the zoom level of the synthetic test images. The mAP is plotted in bins over the zoom level in Figure S9. The behavior is as expected: All models perform very well with an mAP above 0.97 for all reasonable image sizes. Alike the Faster R-CNN models, we see a drop of performance towards very small and very large images due to edge effects, or limits by the pixels per unit cell respectively. Shown previously by the higher ⟨mAP⟩, the YOLOv8 models performed better on synthetic data than YOLOv5. YOLOv5 falls short for very small and very large objects, underlining the positive effect of the hybrid bounding box approach by YOLOv8.

S15.4 Visual comparison

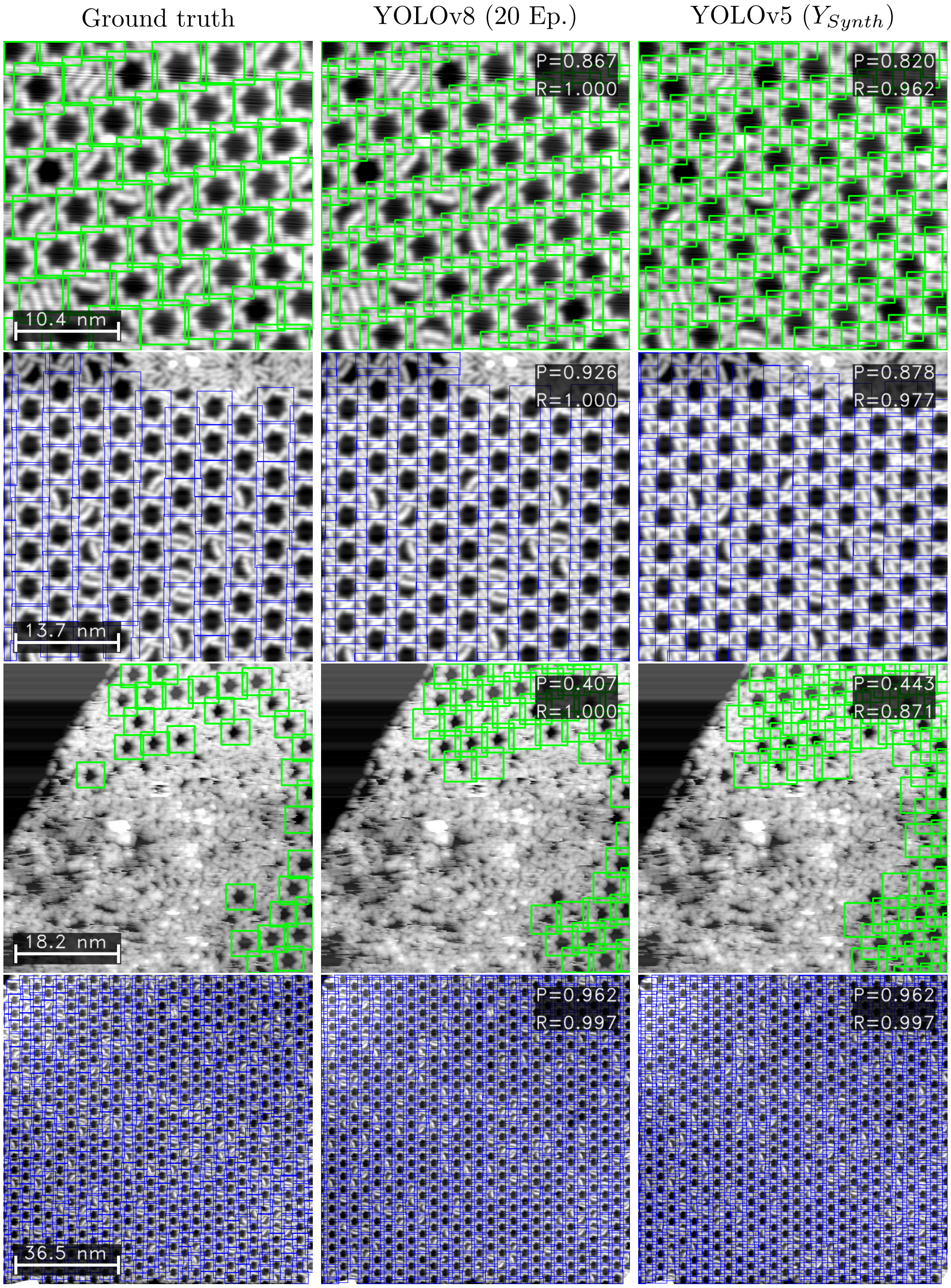

Figure S10: Example results for real images. The first column shows the ground truth (GT), the second column the YOLOv8 model trained on dataset *Synth* for 20 epochs, and the third column the results from stage Y (YOLOv5) using dataset *Synth*. The examples show images of size 31 nm and 41 nm in the first tow rows, a *hard* image with size 55 nm, and a *large* image of size 110 nm. The confidence threshold for all predictions is 0.7.

S16 Orientation of unit-cells

When analyzing physical properties of surfaces, the predicted bounding boxes are a promising first indicator providing information about the density-, spatial- and class distributions along the sample. Having identified individual unit cells automatically furthermore allows to subsequently analyze additional properties of single unit cells. As an example, the orientation of individual unit cells across the scanned image was analyzed.

Since the structure has a six-fold symmetry, the possible alignment angles were defined as $\varphi \in [-30°; 30°)$. The orientation angle was defined as the angle between the orientation of the bottom-left molecule in the unit cell and the image y-axis. With the presented tool for synthetic data generation, reference images of single unit cells with well-defined orientation angles were synthesized. For each chirality, angles were synthesized in 1° steps.

Given the set of reference images, the alignment angle of a single prediction was estimated by first cropping the predicted bounding box, enlarged to equal width and height.

The images in the reference set of the predicted chirality were normalized to the mean and standard deviation of the cropped image. To account for different apparent molecule widths, the reference image was convolved with a Gaussian kernel with the best-matching standard deviation. To find the orientation angle, the mean squared error between the crop and the reference image for each angle was calculated and plotted over the angle. To account for different sized bounding boxes, the similarity was computed for multiple relative scales between the reference and cropped image, and only the highest achieved similarity was taken into account. Inverting and normalizing gives a measure for the similarity over each angle. This plot has a distinct maximum at the angle, at which the reference image matches the cropped image. To obtain this angle, this plot was fitted with a Gaussian curve, whose expectation value and standard deviation give an indicator for the optimal angle and its uncertainty.

Repeating this process for each predicted unit cell in the image, and visualizing the angle by a periodic colormap resulted in a spatial map of alignment angles across the entire STM image. Results are shown in Figure S11.

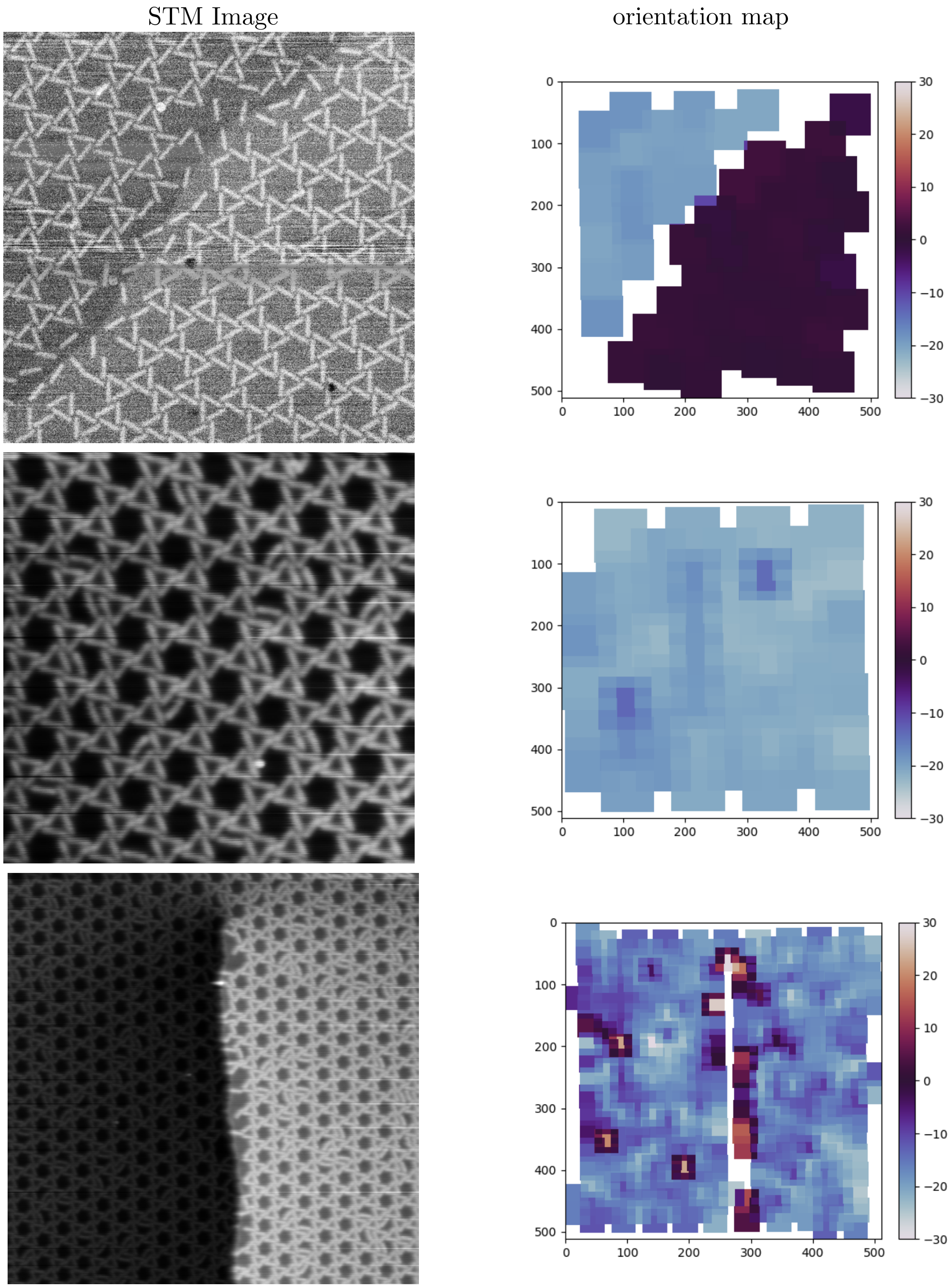

Figure S11: Three examples for finding the orientation of individual unit cells. Left: STM images, right: image colored according to alignment angle of each box. The color shows the estimated angle in degrees. Top row: Synthetic image with atomic step. Second row: plain real image. Third row: real image with dislocation. Outliers occur due to imperfections in the unit cell, or along deformations due to incomplete unit cells.

S17 Example for different structure

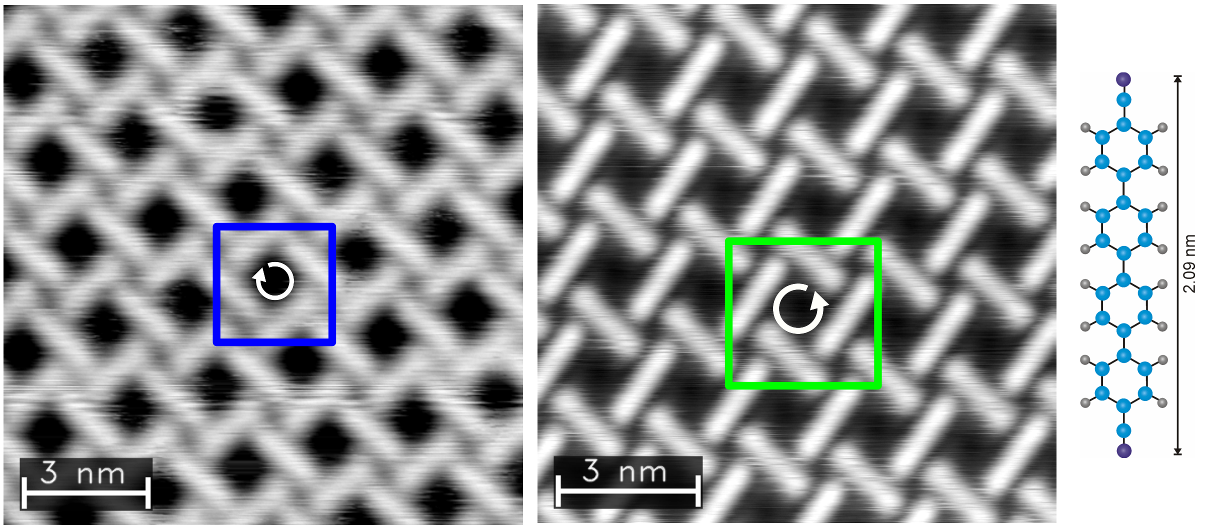

Figure S12: Example STM image and molecular composition of $NCPh_4CN$

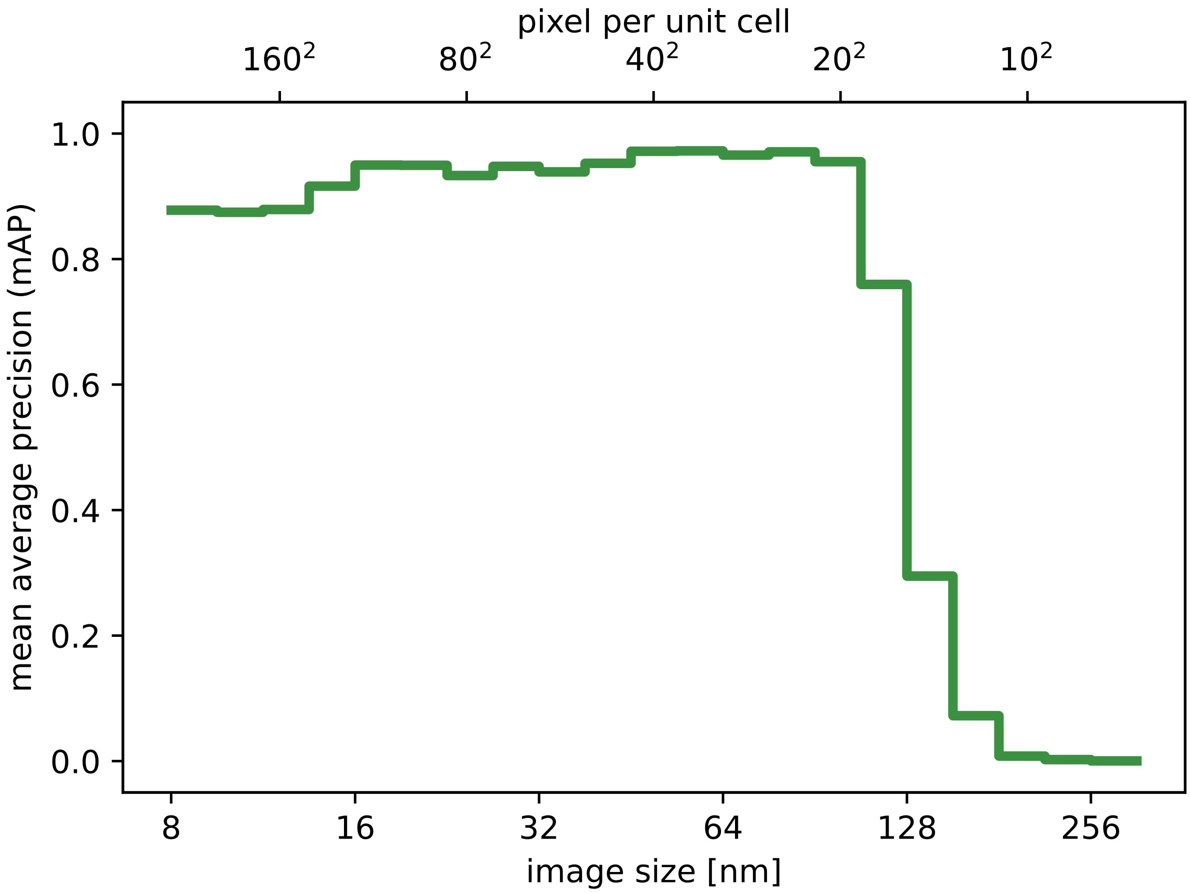

Figure S13: Mean average precision per image size for model Y evaluated on synthetic data of NC$-Ph_4-$CN.

References

[1] L. V. D. Maaten, G. Hinton, *J. Mach. Learn. Res.* **2008,** *9*, 2579.

[2] W. Kohn, A. D. Becke, R. G. Parr, *J. Phys. Chem.* **1996,** *100*, 12974.

[3] J. Pan, *Nat. Comput. Sci.* **2021,** *1*, 95.

[4] N. Kasdin, *Proc. IEEE* **1995,** *83*, 802.

[5] R. Padilla, S. L. Netto, E. A. B. da Silva, In *2020 International Conference on Systems, Signals and Image Processing (IWSSIP)*, Niteroi, Brazil, **2020,** 237.

[6] J. Yan, H. Wang, M. Yan, W. Diao, X. Sun, H. Li, *Remote Sens.* **2019,** *11*, 286.
